# The Prostaglandin E_2_-EP3 Receptor Axis Regulates *Anaplasma phagocytophilum*-Mediated NLRC4 Inflammasome Activation

**DOI:** 10.1371/journal.ppat.1005803

**Published:** 2016-08-02

**Authors:** Xiaowei Wang, Dana K. Shaw, Holly L. Hammond, Fayyaz S. Sutterwala, Manira Rayamajhi, Kari Ann Shirey, Darren J. Perkins, Joseph V. Bonventre, Thangam S. Velayutham, Sean M. Evans, Kyle G. Rodino, Lauren VieBrock, Karen M. Scanlon, Nicholas H. Carbonetti, Jason A. Carlyon, Edward A. Miao, Jere W. McBride, Michail Kotsyfakis, Joao H. F. Pedra

**Affiliations:** 1 Department of Microbiology and Immunology, University of Maryland School of Medicine, Baltimore, Maryland, United States of America; 2 Division of Infectious Diseases, Department of Medicine, Cedars-Sinai Medical Center, Los Angeles, California, United States of America; 3 Department of Microbiology and Immunology, Lineberger Comprehensive Cancer Center, University of North Carolina at Chapel Hill, Chapel Hill, North Carolina, United States of America; 4 Renal Division, Brigham and Women’s Hospital, Department of Medicine, Harvard Medical School, Boston, Massachusetts, United States of America; 5 Department of Pathology, University of Texas Medical Branch, Galveston, Texas, United States of America; 6 Department of Microbiology and Immunology, Virginia Commonwealth University School of Medicine, Richmond, Virginia, United States of America; 7 Institute of Parasitology, Biology Centre, Czech Academy of Sciences, Budweis, Czech Republic; University of São Paulo FMRP/USP, BRAZIL

## Abstract

Rickettsial agents are sensed by pattern recognition receptors but lack pathogen-associated molecular patterns commonly observed in facultative intracellular bacteria. Due to these molecular features, the order *Rickettsiales* can be used to uncover broader principles of bacterial immunity. Here, we used the bacterium *Anaplasma phagocytophilum*, the agent of human granulocytic anaplasmosis, to reveal a novel microbial surveillance system. Mechanistically, we discovered that upon *A*. *phagocytophilum* infection, cytosolic phospholipase A_2_ cleaves arachidonic acid from phospholipids, which is converted to the eicosanoid prostaglandin E_2_ (PGE_2_) via cyclooxygenase 2 (COX2) and the membrane associated prostaglandin E synthase-1 (mPGES-1). PGE_2_-EP3 receptor signaling leads to activation of the NLRC4 inflammasome and secretion of interleukin (IL)-1β and IL-18. Importantly, the receptor-interacting serine/threonine-protein kinase 2 (RIPK2) was identified as a major regulator of the immune response against *A*. *phagocytophilum*. Accordingly, mice lacking COX2 were more susceptible to *A*. *phagocytophilum*, had a defect in IL-18 secretion and exhibited splenomegaly and damage to the splenic architecture. Remarkably, *Salmonella*-induced NLRC4 inflammasome activation was not affected by either chemical inhibition or genetic ablation of genes associated with PGE_2_ biosynthesis and signaling. This divergence in immune circuitry was due to reduced levels of the PGE_2_-EP3 receptor during *Salmonella* infection when compared to *A*. *phagocytophilum*. Collectively, we reveal the existence of a functionally distinct NLRC4 inflammasome illustrated by the rickettsial agent *A*. *phagocytophilum*.

## Author Summary

Elimination of bacteria is orchestrated by the immune system. Intracellular bacteria are generally recognized by cytosolic molecules named Nod-like receptors (NLRs). One such protein scaffold that senses needle-like structures and globular proteins, namely, the bacterial type III secretion (T3SS) and flagellin, is the NLRC4 inflammasome. The NLRC4 inflammasome induces caspase-1 autoproteolysis and secretion of the pro-inflammatory cytokines interleukin (IL)-1β and IL-18. Here, we show that the obligate intracellular rickettsial pathogen *Anaplasma phagocytophilum*, which does not have a T3SS or flagellin-coding genes, induces a distinct NLRC4 inflammasome circuitry through the eicosanoid prostaglandin E_2_ and the EP3 receptor. Conceptually, these findings establish the existence of a distinct microbial surveillance system where the NLRC4 inflammasome senses an obligate intracellular pathogen of public health relevance. Therefore, we propose that rickettsial agents can be used to uncover broader principles of immune surveillance given their unique life style to survive inside the mammalian host and lack of pathogen associated molecular patterns commonly present in most facultative intracellular bacteria.

## Introduction

Rickettsial diseases are arthropod-borne illnesses caused by obligate intracellular bacteria grouped in the order *Rickettsiales* [1, 2]. They include: (*i*) rickettsioses due to bacteria of the genus *Rickettsia*, including the spotted fever and the typhus group; (*ii*) scrub typhus due to *Orientia tsutsugamushi;* and (*iii*) ehrlichioses and anaplasmosis due to bacteria within the family Anaplasmataceae [1, 2]. Some aspects of rickettsial recognition by the immune system have been described [1, 2]. For instance, *Rickettsia* spp. have a structurally distinct form of lipopolysaccharide (LPS) that appears identifiable by Toll-like receptor (TLR)4 [2–5], whereas the TLR2-MyD88 (Myeloid Differentiation Primary Response Protein 88) axis plays a critical role in host defense against ehrlichial infection [6, 7]. However, how these organisms are sensed by pattern recognition receptors (PRRs) remains mostly undefined. *Bona fide* pathogen-associated molecular patterns (PAMPs) are conspicuously absent in some of these microbes when compared to classically-defined bacterial pathogens [2, 8–10]. As an example, *Anaplasma* and *Ehrlichia* spp. are considered Gram-negative bacteria, but are unable to synthesize LPS or peptidoglycans [8, 9, 11]. Additionally, *O*. *tsutsugamushi* does not carry genes in its genome for producing lipid A and has no LPS [10, 12].

Counterintuitively, three independent groups have demonstrated that the NOD (Nucleotide-Binding Oligomerization Domain Protein)-RIPK2 (Receptor-Interacting Serine/Threonine-Protein Kinase 2) pathway, which recognizes peptidoglycans [13], were important to combat *Ehrlichia*, *Anaplasma* and *Orientia* spp. infection [6, 14, 15]. Furthermore, the non-canonical caspase-11 inflammasome, the molecular scaffold that senses LPS in the cytosol and regulates inflammatory cell death or pyroptosis [16], was shown to mediate *Ehrlichia*-induced immunopathology [17]. Nonetheless, *Ehrlichia* spp. do not carry genes for the biosynthesis of LPS in their genomes [11], and are neither cytosolic bacteria nor do they trigger pyroptosis [8]. Mice deficient in NLRC4 [NOD-like receptor (NLR) containing a caspase activating and recruitment domain (CARD) 4], the adaptor molecule that is engaged by NAIP (Neuronal apoptosis inhibitory protein) receptors upon recognition of the bacterial type III secretion system (T3SS) and flagellin [18–24], are also susceptible to *A*. *phagocytophilum* [25]. Importantly, *A*. *phagocytophilum* is aflagellated and does not have a T3SS [9, 26].

These findings suggest that the life style of rickettsial agents induces a mode of immune recognition, which can be exploited for the discovery of unique pathogen-sensing systems. Previously, we discovered that mice deficient in *Nlrc4* and *Caspase-1/11* are susceptible to *A*. *phagocytophilum* infection [25]. We also reported that *A*. *phagocytophilum* causes NLRC4 inflammasome activation and caspase-1 autoproteolysis through the phospholipid-binding protein Annexin A2 [27, 28]. The mechanistic delineation of how the NLRC4 inflammasome was induced remained elusive. In this article, we show a novel mode of NLRC4 inflammasome circuitry that is dependent on the eicosanoid prostaglandin E_2_ (PGE_2_). Upon *A*. *phagocytophilum* infection, cytosolic phospholipase A_2_ (cPLA_2_) cleaves arachidonic acid from phospholipids, which is converted to PGE_2_ via cyclooxygenase 2 (COX2) and membrane associated prostaglandin E synthase-1 (mPGES-1), the terminal enzyme that catalyzes the isomerization of prostaglandin H_2_ (PGH_2_) to PGE_2_ [29, 30]. PGE_2_-EP3 receptor signaling then leads to NLRC4 inflammasome assembly, which induces the release of IL-1β and IL-18. Consistent with our previous reports where mice deficient in RIPK2 are susceptible to *A*. *phagocytophilum* infection [14], we identified RIPK2 as a major regulator of the innate immune response against *A*. *phagocytophilum*. *Ripk2* ^*-/-*^ immune cells exhibited a defect in activation for the nuclear factor (NF)-κB and the NLRC4 inflammasome pathways. Altogether, we define the existence of a functionally distinct NLRC4 inflammasome upon microbial infection.

## Results

### *A*. *phagocytophilum* infection stimulates eicosanoid biosynthesis

**Fig 1:**
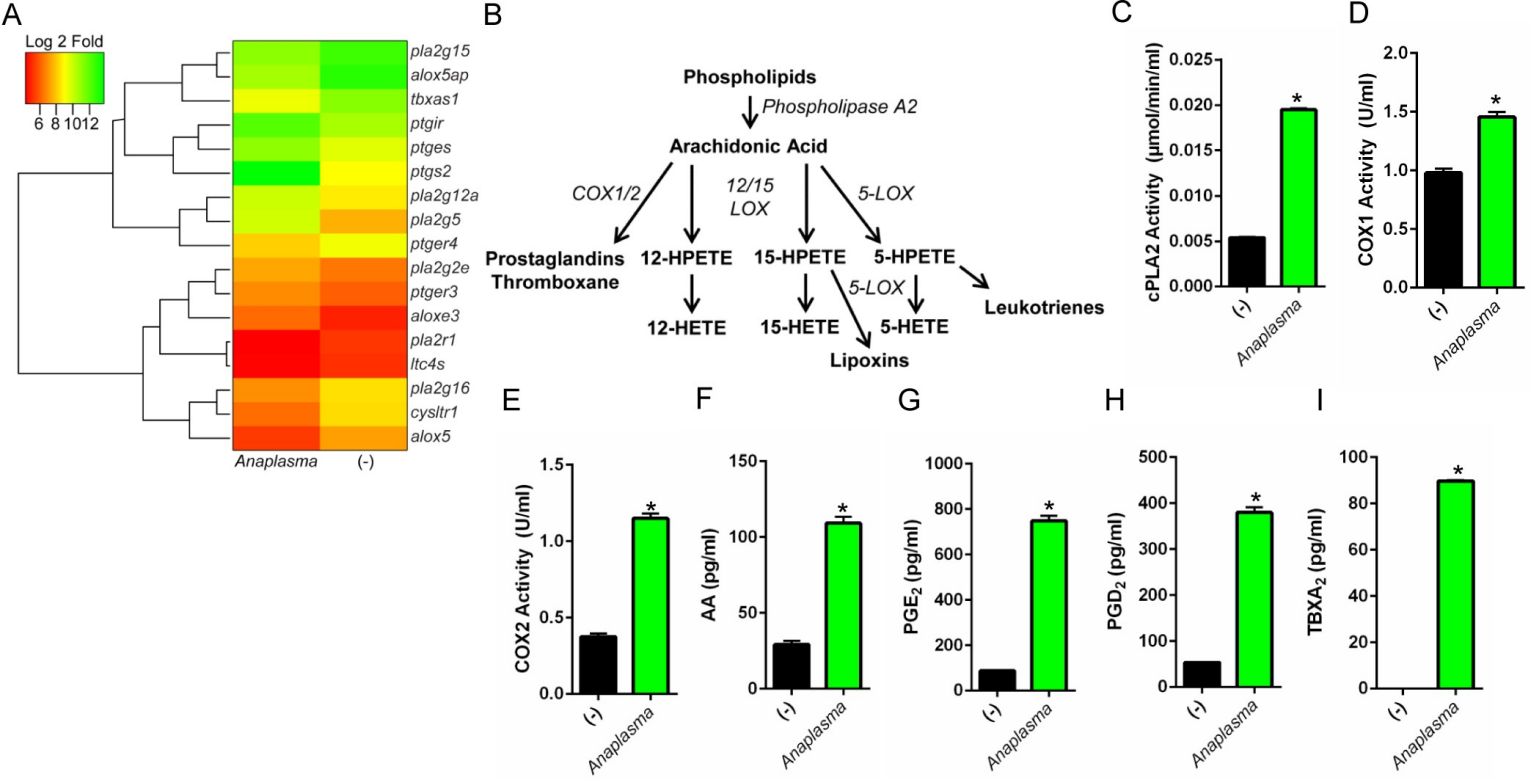
A. phagocytophilum infection induces eicosanoid biosynthesis. (A) Heat map of deep sequencing analysis showing the expression of eicosanoid metabolism genes in murine BMDMs (1.5×10^7^ cells) infected with *A*. *phagocytophilum* (MOI50) for 18 hours. (B) Schematics of eicosanoid metabolism in murine macrophages. Eicosanoid biosynthesis occurs after the release of arachidonic acid from cell membranes by phospholipase A2 (*e*.*g*., cPLA_2_). Arachidonic acid is converted to thromboxane and prostaglandins by cyclooxygenases (COX1/2), whereas 12‐HETE (12‐hydroxyeicosatetraenoic acid), 15‐HETE, 5‐HETE and leukotrienes are synthesized by lipoxygenases (12/15‐LOX and 5-LOX). (C-I) 1.5×10^7^ wildtype (WT) BMDMs were stimulated with *A*. *phagocytophilum* (MOI25) overnight. Cells were scraped followed by sonication. Enzymatic activities of (C) cPLA_2_, (D) COX1 and (E) COX2 were measured. Levels of (F) arachidonic acid (AA), (G) PGE_2_, (H) PGD_2_, and (I) TBXA_2_ in the supernatants of WT BMDMs infected with *A*. *phagocytophilum* (MOI50) were detected. Student’s t test. *P < 0.05. (-) non-stimulated.

*A*. *phagocytophilum* transiently infects bone-marrow derived macrophages (BMDMs) [27, 28] and clinical features in animal models and infected patients suggest classical macrophage activation [31–34]. To determine which genes are important for host immunity, we infected macrophages with *A*. *phagocytophilum*. Deep sequencing analysis [deposited at the Gene Expression Omnibus database (GSE63647)] indicated that the transcription of genes that encode for phospholipase A_2_ (*pla2g12a*, *pla2g5 and pla2g2e*), COX2 (*ptgs2*) and PGE synthase (*ptges*) was increased upon *A*. *phagocytophilum* infection (Fig 1A). These genes are critical for prostanoid biosynthesis (Fig 1B) [35] and correlated with elevated enzymatic activities of cytosolic phospholipase A_2_ (cPLA_2_), COX1 and COX2 (Fig 1C–1E), which led to increased levels of arachidonic acid (AA), PGE_2_, prostaglandin D_2_ (PGD_2_) and thromboxane A_2_ (TBXA_2_) (Fig 1F–1I) upon *A*. *phagocytophilum* infection.

### cPLA_2_ promotes activation of the *A*. *phagocytophilum*-induced NLRC4 inflammasome

**Fig 2:**
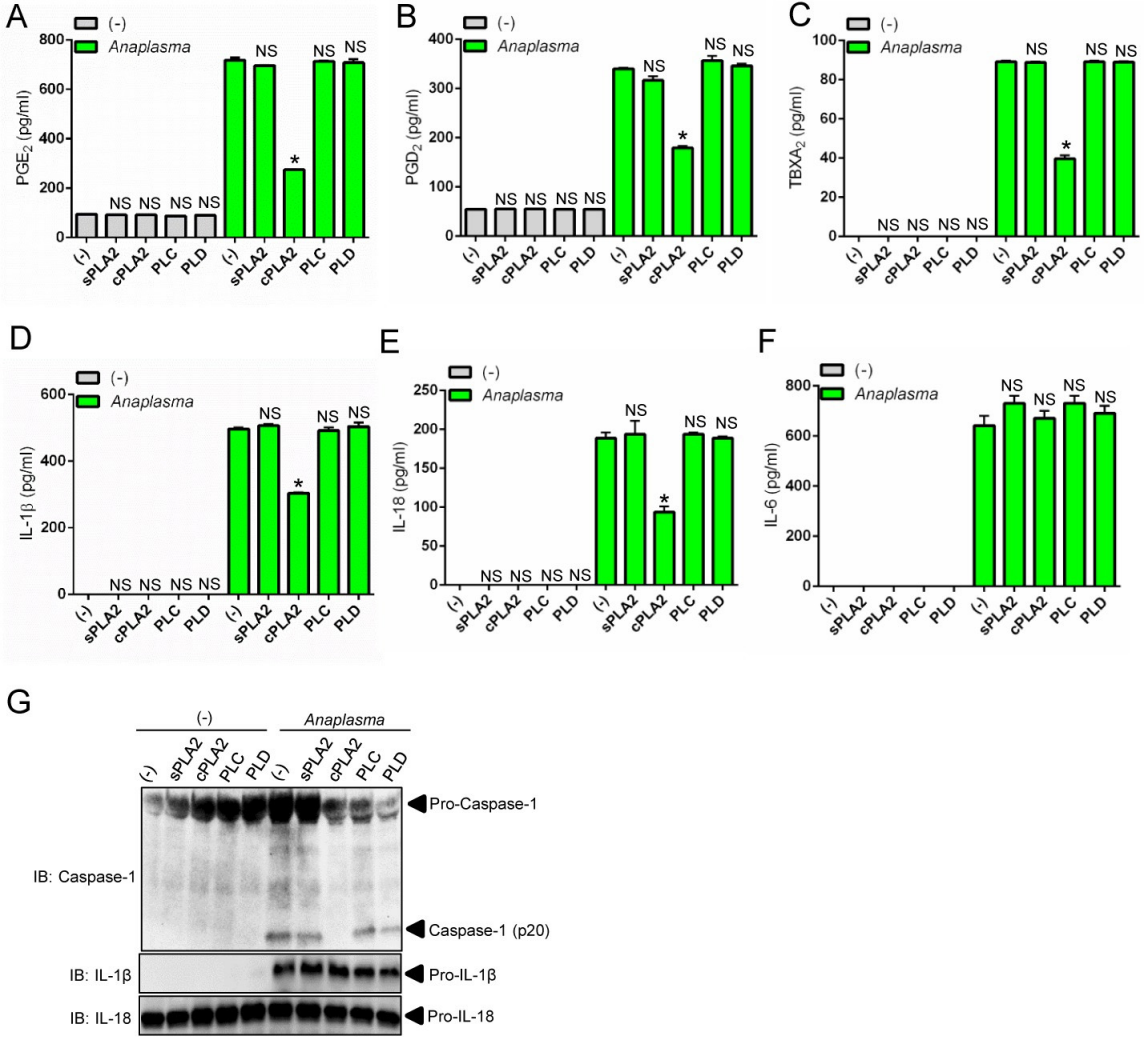
Chemical inhibition of cPLA_2_ affects the *A*. *phagocytophilum*-induced NLRC4 inflammasome. Wildtype (WT) BMDMs (1 x10^6^ cells) pre-treated for 30 minutes with pharmacological inhibitors of secreted PLA_2_ (sPLA_2_) (LY315920–10μM), cPLA_2_ (AACOCF3–10μM), phospholipase C (PLC) (U73122- 10μM) and phospholipase D (PLD) (FIPI– 0.3μM) and infected with *A*. *phagocytophilum* (MOI50) for 18 hours. (A) PGE_2_, (B) PGD_2_, (C) TBXA_2,_ (D) IL-1β, (E) IL-18 and (F) IL-6 levels were measured by ELISA in the cell culture supernatants. (G) SDS-PAGE immunoblot (IB) of caspase-1 p20. pro-IL-1β and pro-IL-18 detected in cell lysates. One way ANOVA-Tukey. *P< 0.05. NS–not significant. (-), non-stimulated.

**Fig 3:**
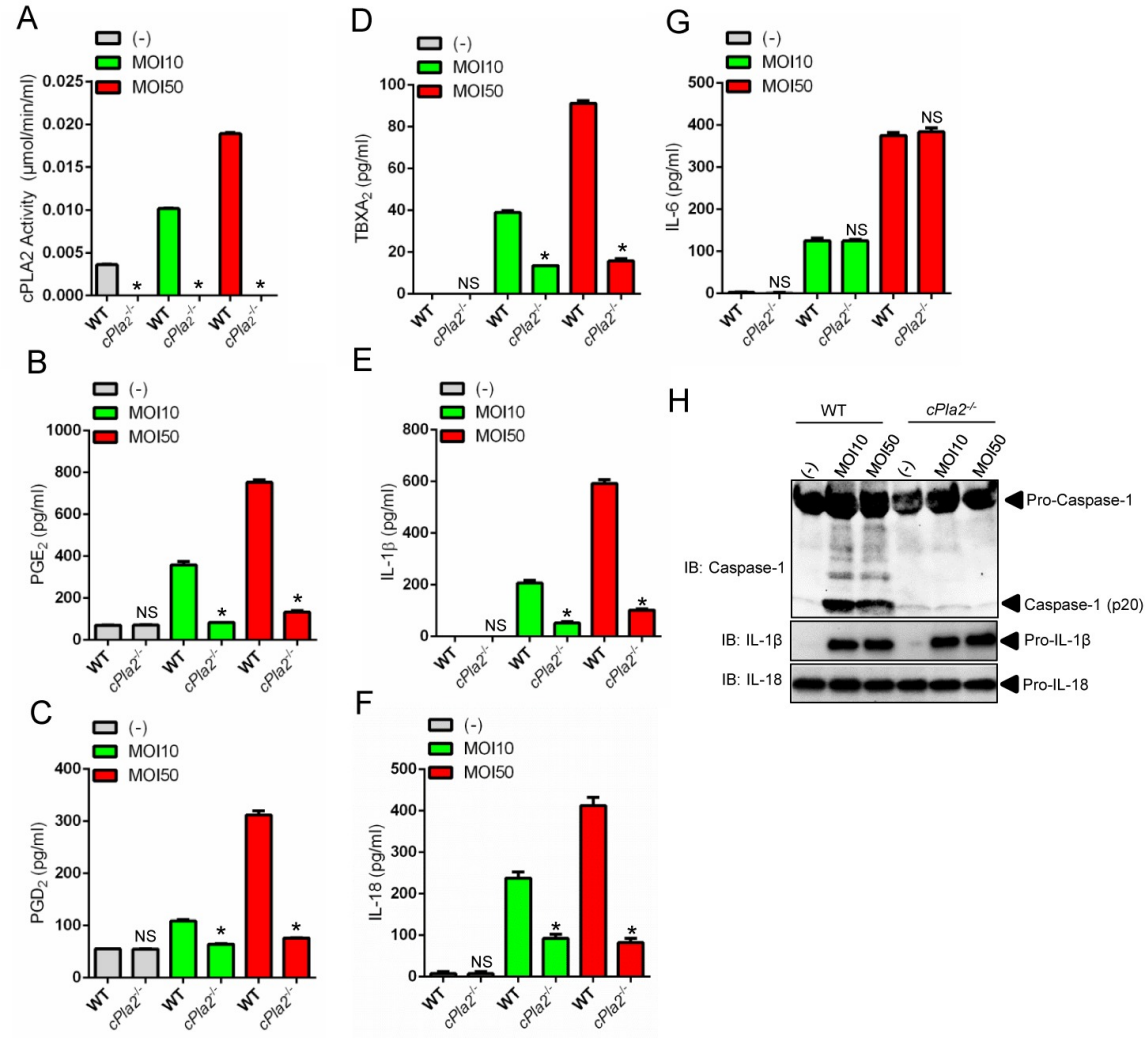
cPLA_2_ regulates the *A*. *phagocytophilum*-induced NLRC4 inflammasome. BMDMs (1 x10^6^ cells) from wildtype (WT) or cPLA_2_-deficient mice were infected with *A*. *phagocytophilum* (MOI 10/50) for 18 hours. (A) cPLA_2_ enzymatic activity was measured. Release of (B) PGE_2_, (C) PGD_2_, (D) TBXA_2,_ (E) IL-1β, (F) IL-18 and (G) IL-6 were measured by ELISA in the cell culture supernatants. (H) SDS-PAGE immunoblot (IB) of caspase-1 (p20) in the supernatants. pro-IL-1β and pro-IL-18 were detected in cell lysates. Student’s t test. *P < 0.05. NS–not significant. (-) non-stimulated.

Eicosanoids have been associated with NLRC4 inflammasome activation [36] and phospholipase A2 releases arachidonic acid from phospholipids for eicosanoid biosynthesis (Fig 1B) [35]. Therefore, we examined whether cPLA_2_ was regulating the *A*. *phagocytophilum*-induced NLRC4 inflammasome. Pharmacological inhibition of cPLA_2_, but not other phospholipases [*e*.*g*., soluble phospholipase A_2_ (sPLA_2_), phospholipase C (PLC) and phospholipase D (PLD)] reduced the levels of PGE_2_, PGD_2_ and TBXA_2_ upon *A*. *phagocytophilum* infection of macrophages (Fig 2A–2C). We also observed lower levels of IL-1β, IL-18 and caspase-1 activation upon bacterial stimulation of immune cells (Fig 2D, 2E and 2G). Similar results were obtained with macrophages deficient in cPLA_2_ at low and high *A*. *phagocytophilum* multiplicity of infection (MOI) (Fig 3A–3F and 3H), indicating that pharmacological inhibition of cPLA_2_ does not lead to off-target effects and the results obtained occurred independently of bacterial numbers. Importantly, secretion of IL-6 and translation of IL-1β and IL-18 by macrophages, which are not regulated by the inflammasome, remained unaffected during pre-treatment of macrophages with pharmacological inhibitors or in the absence of cPLA_2_ (Fig 2F and 2G and Fig 3G and 3H).

Surprisingly, chemical inhibition or genetic ablation of cPLA_2_ did not affect caspase-1 autoproteolysis and cytokine secretion when macrophages were infected with *Salmonella* (S1 Fig), a pathogen that stimulates the NLRC4 inflammasome through the T3SS and flagellin [18–24]. Altogether, these results revealed that although both *A*. *phagocytophilum* and *Salmonella* trigger formation of the NLRC4 inflammasome, the signaling cascades that enable its activation appeared fundamentally different.

### PGE_2_ stimulates assembly of the *A*. *phagocytophilum*-induced NLRC4 inflammasome

**Fig 4:**
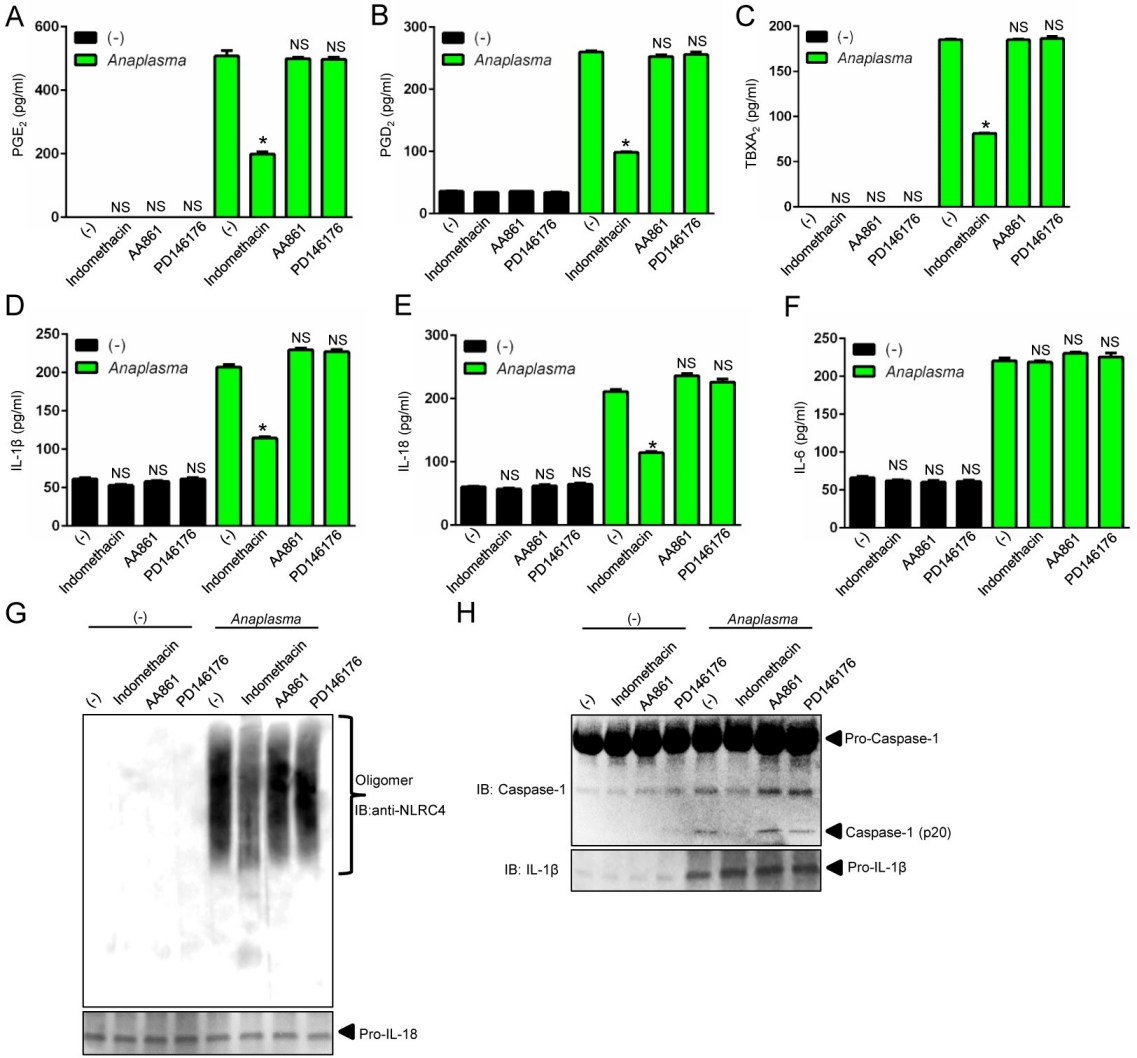
The COX pathway mediates the *A*. *phagocytophilum*-induced NLRC4 inflammasome. Wildtype (WT) BMDMs (1 x10^6^ cells) were pre-treated with indomethacin (100 nM), AA861 (1μg/ml) and PD146176 (1μg/ml) for 2 hours followed by *A*. *phagocytophilum* infection (MOI50) for 18 hours. The levels of (A) PGE_2_, (B) PGD_2_, (C) TBXA_2,_ (D) IL-1β, (E) IL-18 and (F) IL-6 were measured by ELISA in the cell culture supernatants. (G) NLRC4 detection by native gel/immunoblotting (IB) and (H) SDS-PAGE caspase-1 western blot indicating autoproteolysis (p20). Pro-IL-1β and IL-18 were used as loading controls. One-way ANOVA-Tukey *P < .05; NS, not significant. (-) non-stimulated.

**Fig 5:**
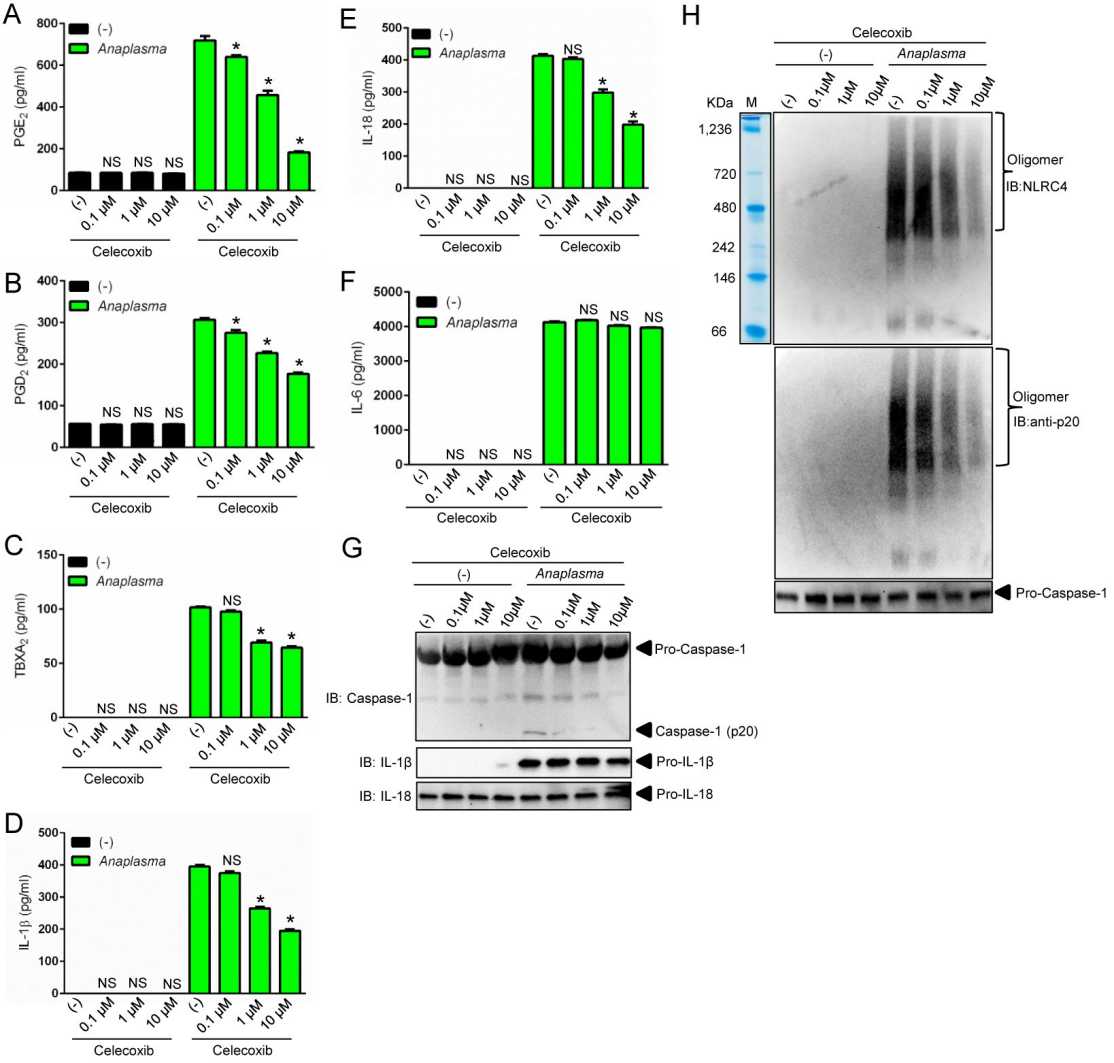
COX2 regulates the *A*. *phagocytophilum*-induced NLRC4 inflammasome. Wildtype (WT) BMDMs (1 x10^6^ cells) were pre-treated with celecoxib (0.1μM to 10 μM) for 2 hours followed by *A*. *phagocytophilum* infection (MOI50) for 18 hours. The levels of (A) PGE_2_, (B) PGD_2_, (C) TBXA_2,_ (D) IL-1β, (E) IL-18 and (F) IL-6 were measured by ELISA in the cell culture supernatants. (G) SDS-PAGE/Western blot (IB) indicating caspase-1 autoproteolysis (p20). (H) NLRC4 inflammasome oligomer detection in the supernatants by native gel/immunoblotting (IB). One-way ANOVA-Tukey *P < .05; NS, not significant. (-) non-stimulated.

**Fig 6:**
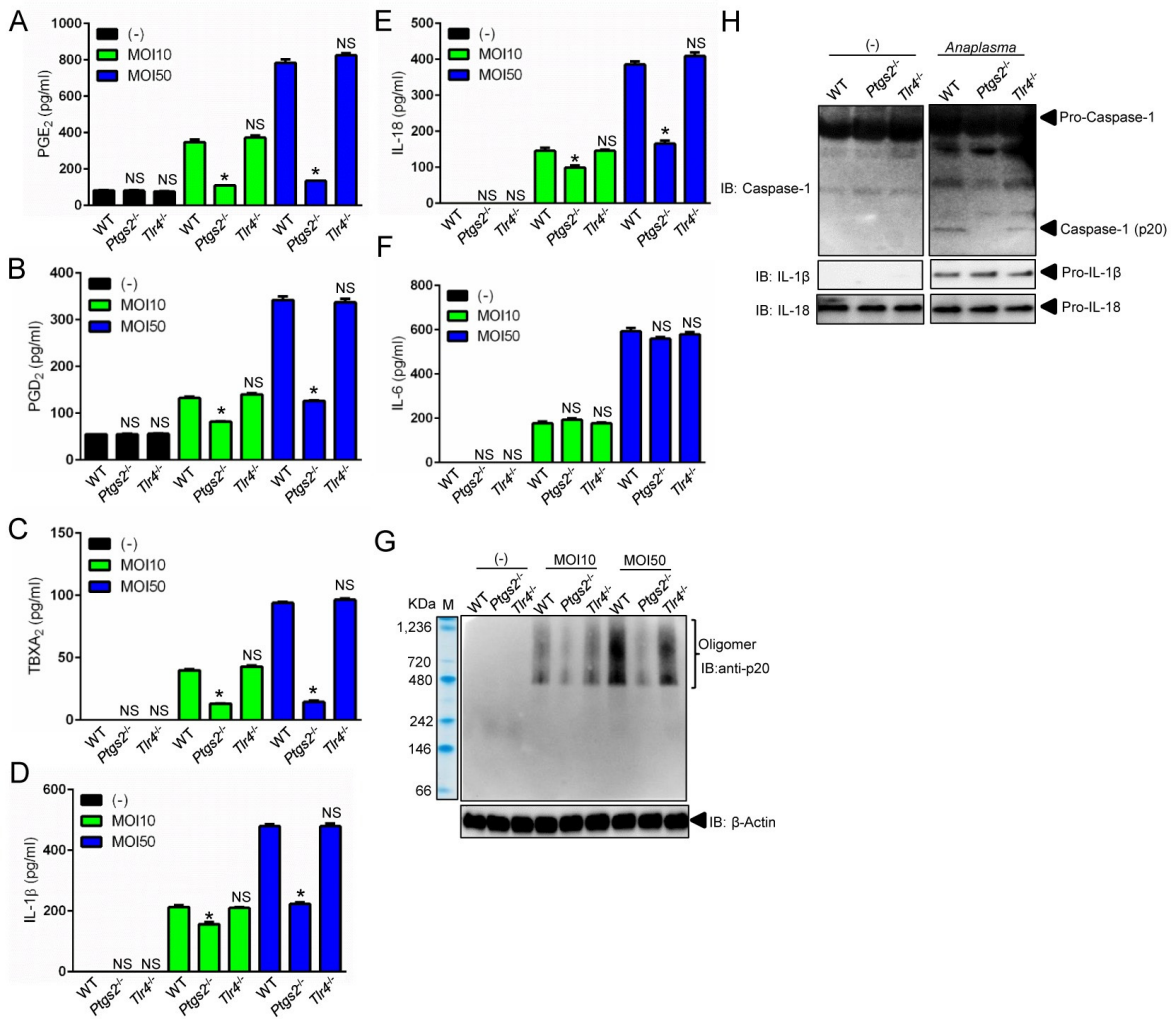
COX2 modulates NLRC4 inflammasome activity upon *A*. *phagocytophilum* infection. BMDMs (1 x10^6^ cells) from wildtype (WT), *Tlr4*- and COX2 (*Ptgs2*)-deficient mice were infected with *A*. *phagocytophilum* (MOI 10/50) for 18 hours. The levels of (A) PGE_2_, (B) PGD_2_, (C) TBXA_2,_ (D) IL-1β, (E) IL-18 and (F) IL-6 were measured by ELISA in the cell culture supernatants. (G) Caspase-1 (p20) native gel and (H) immunoblotting (IB). β-Actin, pro-IL-1β and pro-IL-18 detected in lysates. One way ANOVA-Tukey. *P< 0.05. NS–not significant. (-) non-stimulated.

To gain better insights into the *A*. *phagocytophilum*-induced NLRC4 inflammasome pathway, we pre-treated macrophages with the pan-COX inhibitor indomethacin [37]. Pre-treatment of cells with indomethacin followed by *A*. *phagocytophilum* infection decreased the release of PGE_2_, PGD_2_, TBXA_2_, secretion of IL-1β and IL-18, NLRC4 oligomerization and caspase-1 activation, but not IL-6 secretion by macrophages (Fig 4). To the contrary, pharmacological inhibition of lipoxygenase enzymes, 12/15-LOX (PD146176) or 5-LOX (AA861), did not affect any of the parameters measured (Fig 4). Next, we pre-treated cells with celecoxib, a highly selective COX2 inhibitor [38], followed by *A*. *phagocytophilum* infection. Pre-treatment of wildtype macrophages with celecoxib or, alternatively, *A*. *phagocytophilum* infection of COX2 (*Ptgs2*)-deficient macrophages blunted the release of prostanoids, IL-1β and IL-18, but not IL-6 secretion (Fig 5A–5F and Fig 6A–6F). *A*. *phagocytophilum* infection of COX2 (*Ptgs2*)-deficient macrophages and celecoxib inhibition of COX2 also decreased NLRC4 oligomerization and caspase-1 activation upon *A*. *phagocytophilum* infection (Fig 5G and 5H and Fig 6G and 6H). As expected, no effect was observed for TLR4-deficient macrophages (Fig 6), as *A*. *phagocytophilum* does not carry genes for the biosynthesis of LPS in its genome [9]. Strikingly, *Salmonella* infection or nigericin stimulation of the NLRP3 inflammasome in COX2 (*Ptgs2*)-deficient macrophages had no effect on the release of IL-1β, IL-18, IL-6, inflammasome oligomerization or caspase-1 activation (S2B–S2F Fig). Secretion of PGE_2_ served as positive control for this experiment (S2A Fig).

### *A*. *phagocytophilum*-induced NLRC4 inflammasome activation is coupled to the PGE_2_-EP3 receptor

**Fig 7:**
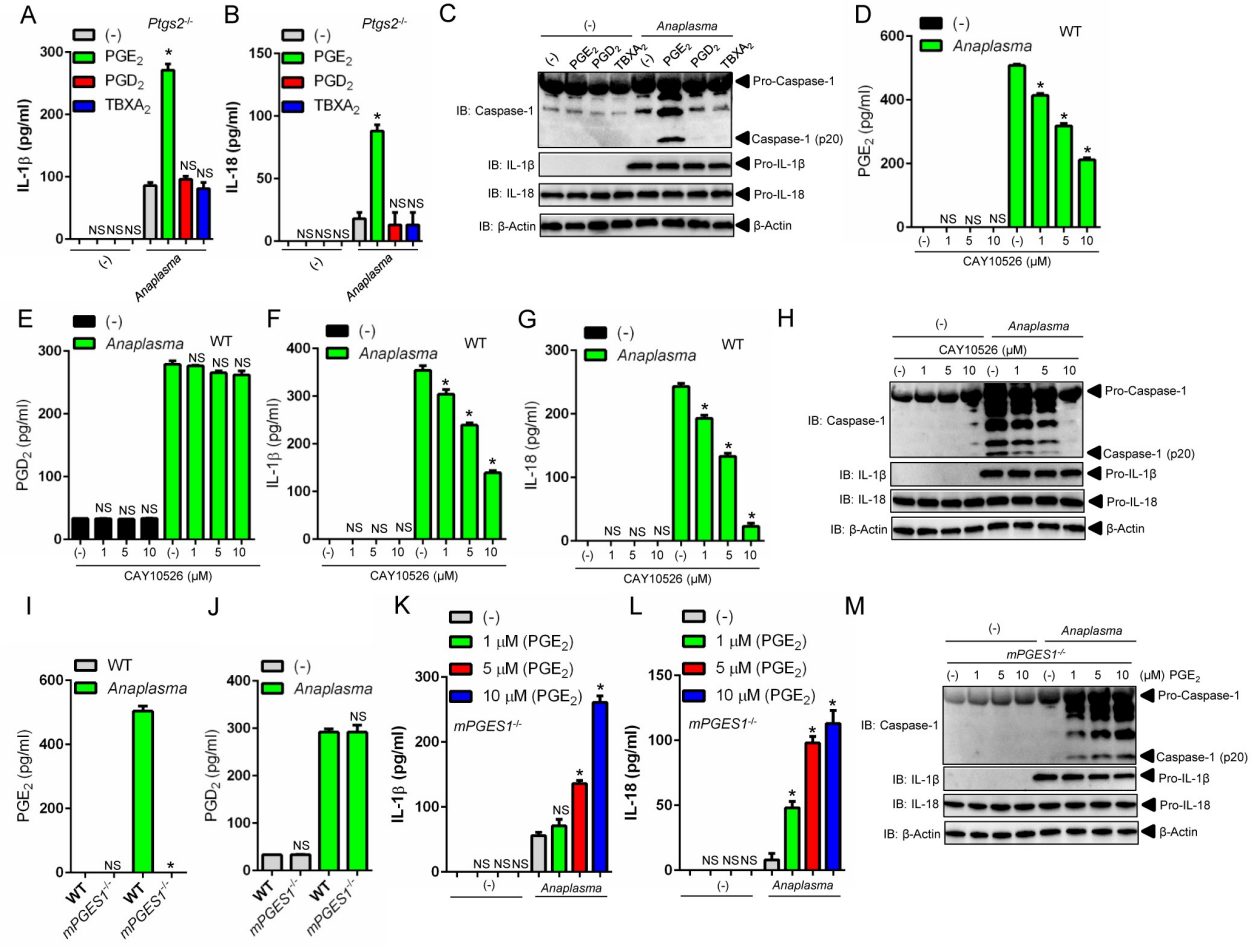
PGE_2_ activates the *A*. *phagocytophilum*-induced NLRC4 inflammasome. (A-C) *Ptgs2* ^-/-^ BMDMs (1 x10^6^ cells) were infected with *A*. *phagocytophilum* (MOI50) for 4 hours followed by addition of PGE_2_ (10 μM), PGD_2_ (10 μM) or TBXA_2_ (10 μM) for 18 hours. (A) Levels of IL-1β and (B) IL-18 in supernatants were measured by ELISA. (C) Caspase-1 autoproteolysis was measured in supernatants of infected cells. pro-IL-1β, pro-IL-18 and β-actin were detected in cell lysates with SDS-PAGE immunoblot (IB). (D-H) Wildtype (WT) BMDMs (1 x10^6^ cells) were pre-treated with the specific inhibitor of mPGES1 (CAY10526–1 μM) for 30 minutes followed by *A*. *phagocytophilum* infection (MOI50) for 18 hours. The levels of (D) PGE_2_, (E) PGD_2_, (F) IL-1β and (G) IL-18 in the culture supernatants were measured by ELISA. (H) Caspase-1 p20 autoproteolysis in culture supernatants. pro-IL-1β, pro-IL-18 and β-actin were detected in cell lysates with a SDS-PAGE immunoblot. (I-J) WT and *mPGES1* ^-/-^ BMDMs (1 x10^6^ cells) were infected with *A*. *phagocytophilum* (MOI50) for 18 hours. The levels of (I) PGE_2_ and (J) PGD_2_ were measured in culture supernatants by ELISA. (K-M) *mPGES1* ^-/-^ BMDMs (1 x10^6^ cells) were infected with *A*. *phagocytophilum* for 4 hours followed by addition of PGE_2_ at indicated concentrations for 18 hours. (K) IL-1β and (L) IL-18 levels in cell culture supernatants were measured by ELISA. (M) Caspase-1 autoproteolysis in cell culture supernatants. pro-IL-1β, pro-IL-18 and β-actin in cell lysates were detected with SDS-PAGE immunoblot (IB). One-way ANOVA-Tukey and Student’s t test; *P < .05; NS, not significant. (-) non-stimulated.

The enzymatic activity of COX2 leads to the biosynthesis of prostanoids [38]. To determine which prostanoid affected the *A*. *phagocytophilum*-induced NLRC4 inflammasome, we performed a multi-pronged approach that included pharmacological inhibition, “add-back” assays and gene-targeted deletion of the membrane associated prostaglandin E synthase-1 (mPGES-1), the terminal enzyme that catalyzes the isomerization of PGH_2_ to PGE_2_ [29, 30]. First, we observed that addition of PGE_2_ in macrophages deficient for *ptgs2* (COX2) restored caspase-1 function and IL-1β and IL-18 secretion upon *A*. *phagocytophilum* infection (Fig 7A–7C). Conversely, the prostanoids PGD_2_ and TBXA_2_ did not elicit the activation of the NLRC4 inflammasome in the presence of *A*. *phagocytophilum* (Fig 7A–7C). Second, specific pharmacological inhibition of the terminal PGE_2_ synthase enzyme, mPGES1 [29, 30], led to reduced caspase-1 activation and IL-1β and IL-18 secretion upon *A*. *phagocytophilum* infection in a dose-dependent manner (Fig 7D–7H). Third, PGE_2_ “add-back” assays restored the phenotype in *mPGES1* ^-/-^ macrophages during *A*. *phagocytophilum* infection (Fig 7I–7M). Importantly, secretion of IL-6 and translation of IL-1β and IL-18 by macrophages, which are not regulated by the inflammasome, remained unaffected during pharmacological inhibition, “add-back” and gene-targeted deletion assays (Fig 7C, 7H and 7M and S3 Fig). Collectively, we provide convincing evidence that PGE_2_ is the sole eicosanoid that induces the activation of the NLRC4 inflammasome upon *A*. *phagocytophilum* infection.

### RIPK2 elicits NLRC4 inflammasome activity during *A*. *phagocytophilum* infection

**Fig 8:**
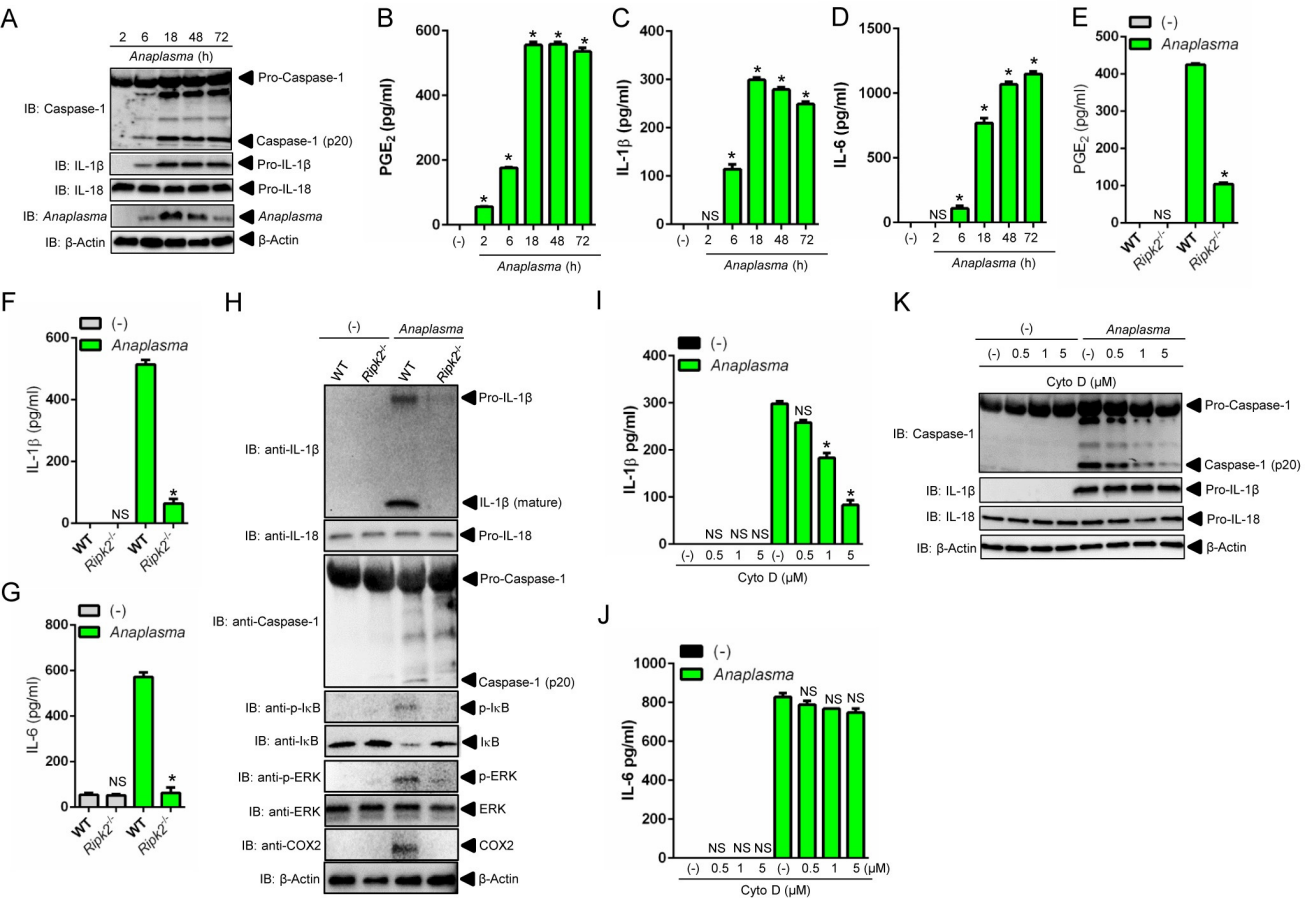
NLRC4 inflammasome activation is dependent on *A*. *phagocytophilum* internalization and RIPK2 function in macrophages. (A-D) Wildtype (WT) BMDMs (1 x10^6^ cells) were infected with *A*. *phagocytophilum* (MOI 50). Cell culture supernatants and lysates were collected at indicated time points post-infection. (A) Caspase-1 autoproteolysis was detected in cell culture supernatants, whereas the presence of *A*. *phagocytophilum*, pro-IL-1β, pro-IL-18 and β-actin are shown in cell lysates with SDS-PAGE immunoblots (IB). The levels of (B) PGE_2_, (C) IL-1β and (D) IL-6 were measured in cell culture supernatants by ELISA. (E-G) BMDMs from WT and *Ripk2* ^-/-^ mice were infected with *A*. *phagocytophilum* (MOI50) (1 x10^6^ cells) for 18 hours. The levels of (E) PGE_2_, (F) IL-1β and (G) IL-6 were measured in cell culture supernatants by ELISA. (H) Caspase-1 autoproteolysis was measured in cell culture supernatants, whereas pro-IL-1β, pro-IL-18, p-IκB, IκB, p-ERK, ERK, COX2 and β-actin were detected in cell lysates by immunoblotting (IB). (I-K) WT BMDMs (1 x10^6^ cells) were pre-treated with indicated concentrations of cytochalasin D for 30 minutes followed by *A*. *phagocytophilum* infection (MOI50) for 18 hours. The levels of (I) IL-1β and (J) IL-6 in cell culture supernatants were measured by ELISA. (K) Caspase-1 autoproteolysis was detected in cell culture supernatants. pro-IL-1β, pro-IL-18 and β-actin were detected in cell lysates with SDS-PAGE immunoblot (IB). One-way ANOVA-Tukey and Student’s t test. *P < .05; NS, not significant. (-) non-stimulated.

Next, we performed a kinetics experiment in macrophages to better characterize *A*. *phagocytophilum* infection in the context of NLRC4 inflammasome biology. As previously shown, *A*. *phagocytophilum* was undetectable inside macrophages at 2-hours post-infection [27]. A small number of bacteria was observed at 6 hours, followed by an increased load at 18 hours and reduction at 48 hours, which led to almost complete elimination after 72 hours of infection in macrophages (Fig 8A) [27]. Consistently, PGE_2_ secretion, caspase-1 activation and IL-1β and IL-18 secretion but not IL-6, peaked at 18 hours, the same time point where the greatest number of *A*. *phagocytophilum* was detected inside macrophages (Fig 8A–8D and S4A Fig).

*A*. *phagocytophilum* does not synthesize LPS or peptidoglycans [8, 9, 11]. Therefore, one interesting immunological question pertains to the host molecule that induces NF-κB activation upon infection. We reasoned that RIPK2 could be this master regulator. This hypothesis rested on four findings. First, RIPK2 activates NF-κB signaling and mitogen activated protein (MAP) kinases upon infection [13]. Second, *A*. *phagocytophilum* interacts with the host endoplasmic reticulum (ER) [39], which may exert RIPK2 activity in the absence of peptidoglycans due to cellular stress [40]. Third, COX2 expression is regulated through a signaling cascade that converges at the MAP kinase and the NF-κB pathways [41]. Fourth, mice deficient in RIPK2 are susceptible to *A*. *phagocytophilum* infection and secrete reduced levels of IL-18 in the peripheral blood [14]. Accordingly, *ripk2* ^*-/-*^ macrophages exhibited a defect in NF-κB and MAP kinase signaling, which led to decreased translation of COX2, pro-IL-1β and IL-6 secretion (Fig 8G and 8H). RIPK2 activity also affected PGE_2_ release and caspase-1 autoproteolysis upon *A*. *phagocytophilum* infection, as indicated by reduced levels of PGE_2,_ IL-1β, IL-18 and caspase-1 activation in cell culture supernatants of *ripk2* ^*-/-*^ macrophages (Fig 8E, 8F and 8H and S4B Fig). Finally, *A*. *phagocytophilum* internalization was important for PGE_2_ release and NLRC4 inflammasome activation, as demonstrated in our experiments with cytochalasin D, a potent mycotoxin that inhibits actin polymerization (Fig 8I–8K and S4C and S4D Fig). Collectively, we identified RIPK2 as a major regulator of the innate immune response against *A*. *phagocytophilum*.

### The PGE_2_-EP3 receptor regulates activation of the NLRC4 inflammasome upon *A*. *phagocytophilum* infection

**Fig 9:**
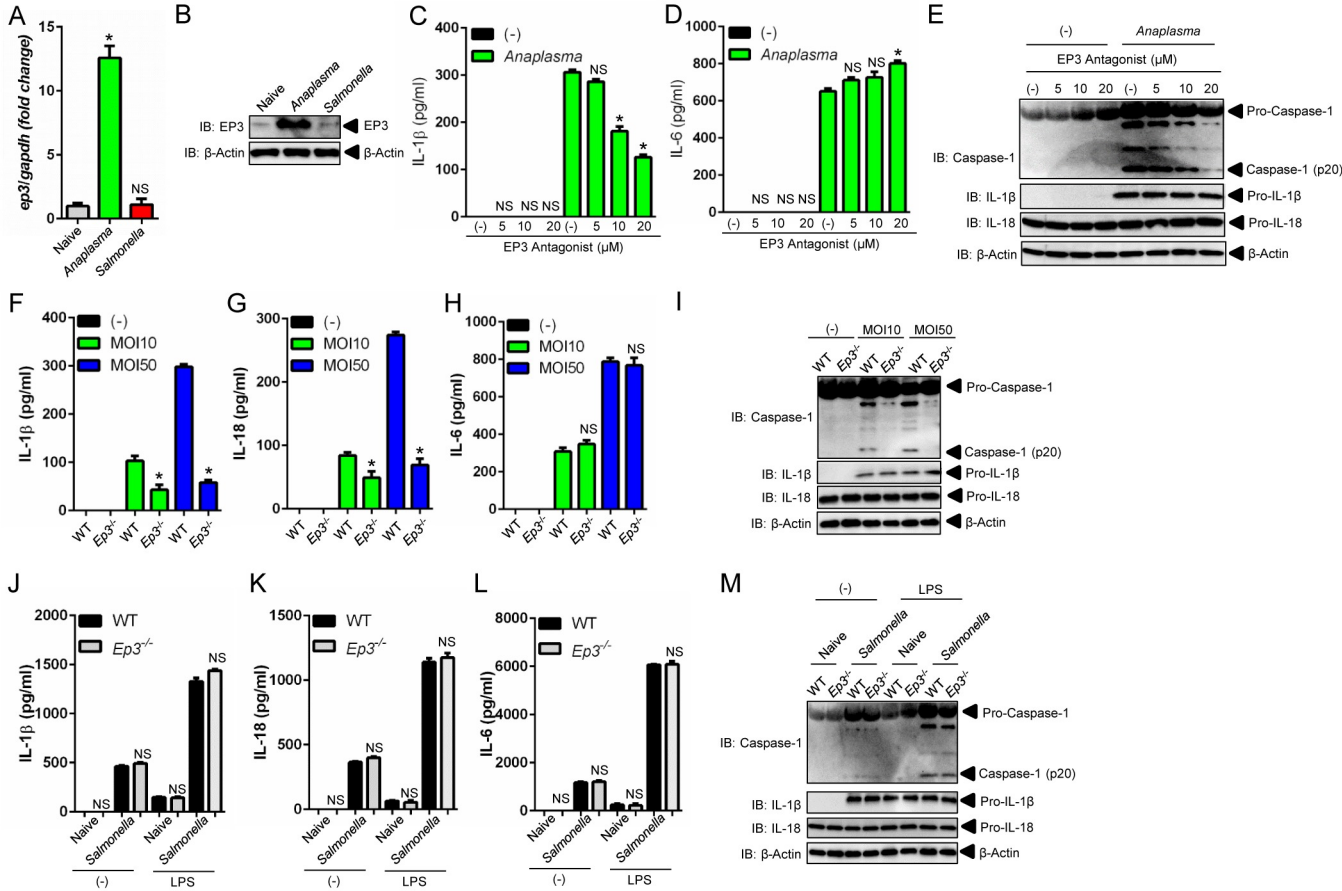
The PGE_2_-EP3 axis regulates activation of the NLRC4 inflammasome upon *A*. *phagocytophilum* infection. (A-B) Wildtype (WT) BMDMs (1 x10^6^ cells) were infected with *A*. *phagocytophilum* (MOI50) for 18 hours or *Salmonella* (MOI25) for 1 hour. RNA and protein levels of the PGE_2_-EP3 receptor was measured by qRT-PCR and western blot (IB). β-actin was also detected in cell lysates. (C-E) WT BMDMs (1 x10^6^ cells) was pre-treated with the EP3 antagonist (L-798106) for 30 minutes at indicated concentrations followed by *A*. *phagocytophilum* colonization (MOI50) for 18 hours. The levels of (C) IL-1β and (D) IL-6 were measured in cell culture supernatants by ELISA. (E) Caspase-1 autoproteolysis in cell culture supernatants. pro-IL-1β, pro-IL-18 and β-actin were detected in cell lysates by SDS-PAGE immunoblots (IB). (F-H) WT and *Ep3* ^-/-^ BMDMs (1 x10^6^ cells) were infected with *A*. *phagocytophilum* (MOI 10/50) for 18 hours. The levels of (F) IL-1β, (G) IL-18 and (H) IL-6 in culture supernatants were measured by ELISAs. (I) Caspase-1 autoproteolysis in cell culture supernatants. pro-IL-1β, pro-IL-18 and β-actin were detected in cell lysates by SDS-PAGE immunoblots (IB). (J-M) Naïve or LPS-primed (50 ng/ml) WT and *Ep3* ^-/-^ BMDMs (1 x10^6^ cells) were infected with *Salmonella* (MOI25) for 1 hour. The levels of (J) IL-1β, (K) IL-18 and (L) IL-6 in cultured supernatants were measured by ELISA. (M) Caspase-1 autoproteolysis in cell culture supernatants. pro-IL-1β, pro-IL-18 and β-actin were detected in cell lysates by SDS-PAGE immunoblots (IB). Student’s t test and ANOVA-Tukey. *P < .05; NS, not significant. (-) non-stimulated.

We then blunted the PGE_2_ signaling cascade with chemical antagonists that bind covalently to the four PGE_2_ receptor subtypes (EP1-EP4) [30] and compared our findings with *Salmonella*. We observed that inhibition of the PGE_2_-EP3 receptor significantly decreased IL-1β and IL-18 release, and caspase-1 activation, but not IL-6 secretion upon *A*. *phagocytophilum* infection (Fig 9C–9E and S5 Fig). The EP3 receptor for PGE_2_ is sensitive to pertussis toxin (PT) [42]. Macrophages pre-treated with PT and then stimulated with *A*. *phagocytophilum* also resulted in inhibition of the NLRC4 inflammasome (S5A–S5D Fig). Importantly, the catalytically inactive pertussis toxin (PT*), with a two amino acid substitution (9K129G) [43], did not block PGE_2_ signaling upon *A*. *phagocytophilum* colonization (S5A–S5D Fig). Next, we took advantage of the *ep3* ^-/-^ mice and showed that in the absence of the EP3 receptor molecule, *A*. *phagocytophilum* did not induce caspase-1 activation and IL-1β and IL-18 secretion by macrophages (Fig 9F–9I). Conversely, lack of the PGE_2_-EP3 receptor did not affect the NLRC4 inflammasome induced by *Salmonella* (Fig 9J–9M and S6 Fig).

PGE_2_ exerts its actions by acting on G-protein-coupled receptors (GPCRs). PGE_2_ binds to the EP3 receptor, which inhibits the membrane associated adenylyl cyclase via Gαi [44]. This signaling relay decreases cytosolic cyclic AMP (cAMP) production, as adenylyl cyclase catalyzes the conversion of adenosine triphosphate (ATP) to cAMP [44] (S8 Fig). We validated these observations with sulprostone, an EP3 agonist and positive control in our assays (S7A Fig). Consistently, *A*. *phagocytophilum* colonization of macrophages led to reduced production of cAMP (S7A Fig). Moreover, pharmacological blockade of the PGE_2_-EP3 receptor via the EP3 antagonist or PT hindered the inhibition of cAMP by *A*. *phagocytophilum* (S7A Fig).

Next, we showed that membrane, but not soluble, adenylyl cyclase modulated the *A*. *phagocytophilum*-induced NLRC4 inflammasome. Forskolin, a selective inhibitor of the membrane-associated adenylyl cyclase [45], inhibited IL-1β, IL-18 and caspase-1 autoproteolysis during *A*. *phagocytophilum* infection of macrophages (S7B–S7E Fig). On the other hand, pre-treatment of macrophages with KH7, a specific pharmacological inhibitor of soluble adenylyl cyclase [45], did not affect NLRC4 inflammasome function during *A*. *phagocytophilum* infection (S7F–S7I Fig). Altogether, these findings: (*i*) indicated that the PGE_2_-EP3 axis is critical for the NLRC4 inflammasome elicited by *A*. *phagocytophilum*; and (*ii*) explained why *Salmonella* is unable to trigger a similar pathway when compared to *A*. *phagocytophilum*. This was likely due to reduced expression of the EP3 receptor during *Salmonella* infection of macrophages (Fig 9A and 9B).

### Mice deficient in COX2 are susceptible to *A*. *phagocytophilum*

**Fig 10:**
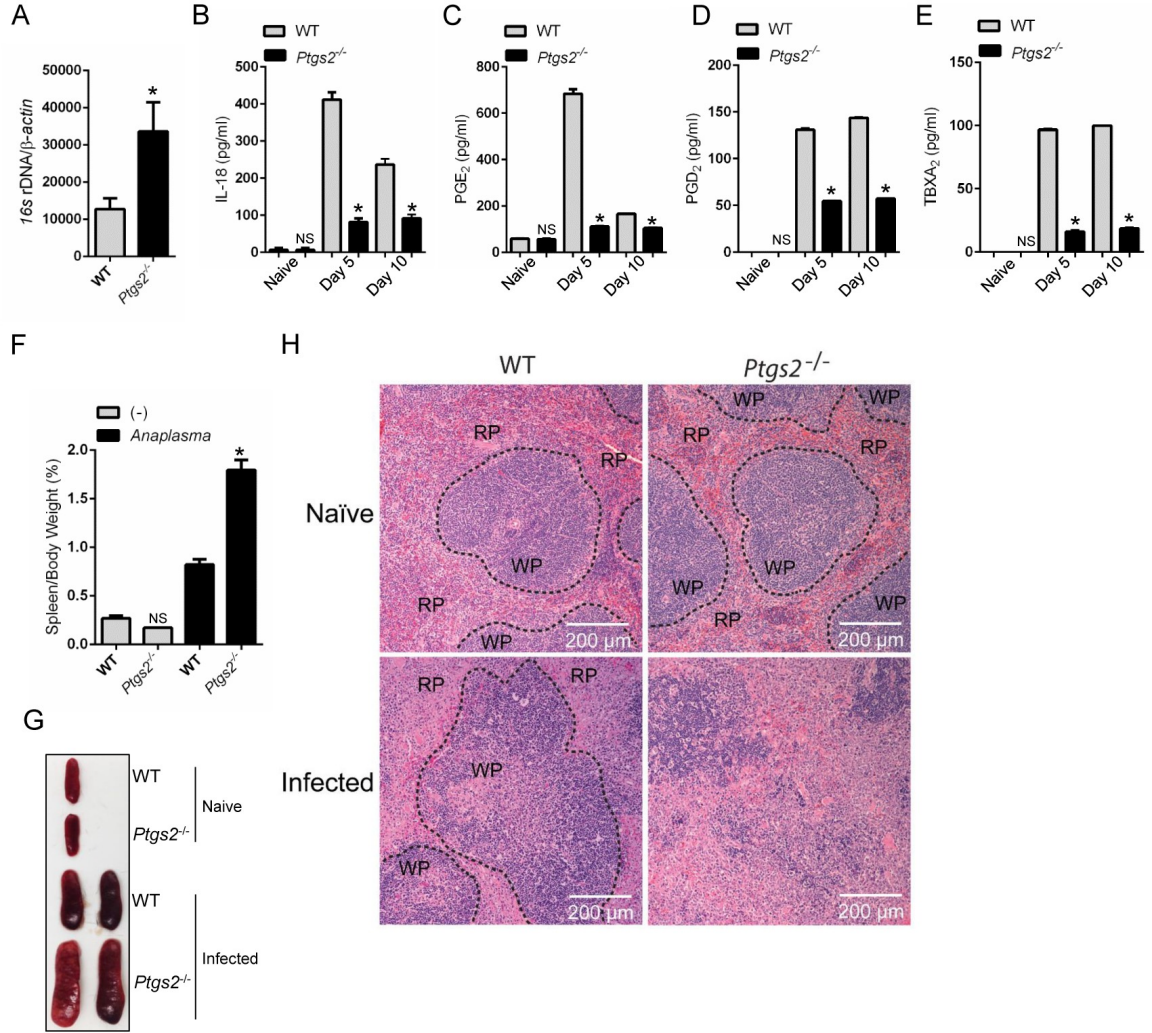
COX2 restricts *A*. *phagocytophilum* infection *in vivo*. *A*. *phagocytophilum* infection of WT (n = 20) and COX2 (*ptgs2)* ^-/-^ (n = 10) mice. Bacterial load in the (A) peripheral blood of infected mice at day 15. (B) IL-18, (C) PGE_2_, (D) PGD_2_ and (E) TBXA_2_ release in the serum of infected animals. (F-G) Splenomegaly for COX2 (*ptgs2* ^-/-^) mice infected with *A*. *phagocytophilum*. (H) Splenic architecture depicting the red (RP) and the white (WP) pulp during *A*. *phagocytophilum* infection. One-way ANOVA-Tukey; Student t test; *P < 0.05. NS–not significant. (-) non-stimulated.

To prove that the results obtained *in vitro* could also be observed *in vivo*, we then infected mice deficient in COX2 (*Ptgs2*) with *A*. *phagocytophilum*. *Ptgs2*-deficient animals were more susceptible to *A*. *phagocytophilum* infection (Fig 10A) and exhibited reduced levels of IL-18 in the peripheral blood when compared to the wildtype mice (Fig 10B). As previously seen, no detectable levels of IL-1β were observed in the blood of *A*. *phagocytophilum*-infected mice [25]. These findings agreed with our prior publications, showing that IL-18 release mediated by RIPK2 and the NLRC4 inflammasome regulates interferon (IFN)-γ production by CD4^+^ T cells upon *A*. *phagocytophilum* infection [14, 25]. COX2 (*Ptgs2*)-deficient mice infected with *A*. *phagocytophilum* also revealed lower levels of PGE_2_, PGD_2_, TBXA_2_ and splenomegaly (Fig 10C–10G). COX2 (*Ptgs2*)-deficient animals had increased cellular infiltration in the red pulp and damage to the splenic architecture upon *A*. *phagocytophilum* infection (Fig 10H). In sum, these results showed that COX2 is critically important for *A*. *phagocytophilum* infection *in vivo*.

## Discussion

The NLRC4 inflammasome is currently thought to only recognize components of the bacterial T3SS and flagellin [18–24]. Other inflammasomes, however, such as the NLRP3 scaffold, sense a wide-range of molecular structures leading to caspase-1 activation and cytokine secretion [16]. We hypothesized that an alternative signaling cascade for the NLRC4 inflammasome must exist because mice deficient in NLRC4 are susceptible to *A*. *phagocytophilum* infection [25], an obligate intracellular rickettsial bacterium that does not have a T3SS and flagellin [11]. Furthermore, *Annexin a2*-deficient mice were more susceptible to *A*. *phagocytophilum* infection and showed splenomegaly, thrombocytopenia and monopenia [28]. Macrophages deficient in Annexin A2, a phospholipid-binding protein, secreted significantly smaller amounts of IL-1β and IL-18 and had a defect in NLRC4 inflammasome oligomerization and caspase-1 activation [28]. In contrast, *Annexin a2* ^-/-^ macrophages released IL-1β, IL-18, and IL-6 at wild-type levels when infected with *Salmonella*, a canonical NLRC4 agonist [28].

We provide unequivocal evidence that two distinct signaling pathways occur for NLRC4 inflammasome activation within the cell: one termed classical (*i*.*e*., stimulated by *Salmonella*) and another referred to as alternative (*i*.*e*. described here, responding to *A*. *phagocytophilum*). Given how inflammasome biology intersects with a growing number of disciplines, we reason that these findings are conceptually valuable because we reveal that eicosanoid receptors in immune cells activate diverging signaling cascades. For instance, both *A*. *phagocytophilum* and *Salmonella* lead to PGE_2_ production by macrophages. However, *Salmonella* is unable to activate the eicosanoid-dependent NLRC4 inflammasome pathway because it does not induce PGE_2_-EP3 receptor expression.

PGE_2_ is likely acting in an autocrine/paracrine manner to drive NLRC4 inflammasome activation upon *A*. *phagocytophilum* infection. This is based on the evidence that *A*. *phagocytophilum* infection upregulates the EP3 receptor, which is known to elicit PGE_2_ signaling in a cell-intrinsic manner [30, 35]. Alternatively, PGE_2_ may also affect the function of “bystander” cells in a paracrine manner given that our exogenous PGE_2_ “add-back” assays restored NLRC4 inflammasome activity in *A*. *phagocytophilum*-infected cells.

Can rickettsial agents be used to uncover broader principles of immune sensing? The answer to this question may have to deal with the biology of these organisms. Rickettsial agents differ greatly in terms of how they invade and replicate within the mammalian host when compared to other bacteria commonly used to study microbial immunity. Their obligate intracellular life style, coupled to the intense selective pressure to survive both in the arthropod vector and the mammalian host [1, 2] suggests that these microbes have to employ extreme measures to conceal themselves from the immune system. This reasoning may explain why *A*. *phagocytophilum* triggers such a distinct pathogen-recognition mechanism when compared to other bacteria.

In summary, we discovered a novel mode of NLRC4 inflammasome activation triggered by the rickettsial bacterium *A*. *phagocytophilum*. We revealed that some microbial pathogens lacking the T3SS and flagellin activate the NLRC4 inflammasome. We also illustrated how this protein scaffold distinguishes bacterial infection within the cell. Altogether, our findings suggest that there are broader yet-to-be discovered principles of microbial sensing in the context of NLRC4 inflammasome biology.

## Materials and Methods

### Mice and bacteria

Breeding and experiments were performed in strict compliance with guidelines set forth by the National Institutes of Health (Office of Laboratory Animal Welfare [OLAW] assurance number A3200-01). Procedures were approved by the Institutional Biosafety (IBC:00002247) and Animal Care and Use (IACUC:0413017 and 0216015) committees at the University of Maryland, Baltimore. *Ripk2* ^-/-^ (007017), C57BL/6 (000664) and *Ptgs2* ^-/-^ (COX2) mice (008101) were purchased from Jackson Laboratories. Femurs from *mPGES1* ^-/-^ [29] and *Ep3* ^-/-^ [46] mice were a gift from Leslie Crofford and Richard Breyer at Vanderbilt University School of Medicine. *Tlr4* ^-/-^ and *cPla2* ^*-/-*^ mice were previously described [47, 48]. Mice were gender matched and at least 6–10 weeks of age. BMDMs were generated, as previously described [27]. Culturing for the *A*. *phagocytophilum* strain HZ and calculations were described elsewhere [27]. *Salmonella* strain SL1344 was a gift from Dr. Stefanie Vogel at the University of Maryland, Baltimore School of Medicine. *Salmonella* was grown in HS media at 37°C and enumerated, as previously described [49]. Cell cultures were tested and determined to be *Mycoplasma*-negative through a commercially available PCR kit (Southern Biotech -13100-01).

### Chemical reagents

LPS (50ng/ml) was purchased from InvivoGen. Nigericin (10μM), indomethacin (100 nM) and celecoxib (0.1μM to 10 μM) were purchased from Sigma-Aldrich. AA861 (1μg/ml) and PD146176 (1μg/ml) were purchased from BioMol International. CAY10526 (10010088), KH7 (13243), Forskolin (11018), Cytochalasin D (11330), PGE_2_ (14010), PGD_2_ (12010) and U46619 (thromboxane A2 analogue, 16450) were purchased from Cayman Chemicals. The PGE_2_ receptor antagonists EP1–1μM (SC51089), EP2–5μM (AH6809) and EP4–5μM (ONO-AE3-208) were purchased from Cayman Chemical, whereas the antagonist for the PGE_2_ EP3–10μM (L-798106) and the PGE_2_ EP3 receptor agonist (sulprostone—3μM) was purchased from Sigma. The inhibitors for the phospholipases cPLA2 (AACOCF3), sPLA2 (LY315920), PLC (U73122) and PLD (FIPI) were purchased from Tocris Bioscience. Pertussis toxin (PT) and the catalytically inactive pertussis toxin (PT*) with a two amino acid substitution (9K129G) were described previously [43, 50].

### Bacterial infection of macrophages

1×10^6^ BMDMs were seeded into 24-well plate in 300 μl of media containing 5% fetal bovine serum (FBS) overnight prior to the challenge by either *A*. *phagocytophilum* (MOI 10 and 50) or *Salmonella* (MOI 25) for 1 hour. 50ng/ml of LPS was used for cell priming at 37°C and 5% CO_2_ for 30 minutes during *Salmonella* infection. LPS-primed cells were washed twice extensively followed by the addition of bacteria. In inhibition assays, 1×10^6^ WT and genotype-deficient BMDMs were pre-treated with pharmacological inhibitors at indicated time and concentrations followed by the stimulation with *A*. *phagocytophilum* (MOI 10 and 50) overnight or *Salmonella* (MOI 25) for 1 hour. For the *Ptgs2* ^-/-^ and *mPGES1* ^-/-^ “add-back” experiments, 1×10^6^ WT and deficient cells were infected with *A*. *phagocytophilum* (MOI 50) for 4 hours followed by the addition of the respective eicosanoid at indicated concentrations for 18 hours. After infection, cultured supernatants and cell lysates collected from each well were used for ELISA and immunoblot assays.

### Native polyacrylamide gel electrophoresis

Equal amounts of supernatants were mixed with the native sample buffer (62.5 mM Tris-HCl, 40% glycerol, 0.01% bromophenol blue, pH 6.8), loaded into 4–15% Mini-PROTEAN TGX Precast Gels and run at 200 volts for 2 hours in 1×Tris/Glycine native running buffer (25 mM Tris, 192 mM glycine, pH8.3). NativeMark Unstained Protein Standard (Invitrogen) was visualized with Gel Code Blue Safe Protein Stain solution (Thermo Scientific).

### Immunoblotting

Cell lysates were prepared in radioimmunoprecipitation (RIPA) lysis buffer (Boston Bioproducts) with Halt Protease Inhibitor Cocktail (Thermo Scientific) and PhosSTOP (Roche Applied Science). 4–15% Mini-PROTEAN TGX precast gels were run at 200 volts for 30 minutes in the 1×Tris-Glycine-SDS running buffer (Boston Bioproducts). Transfer was performed using the Bio-Rad Trans-Blot Turbo with either polyvinylidene fluoride (PVDF) or nitrocellulose membranes (Bio-Rad). Membranes were blocked in 5% skim milk or BSA (Bio-Rad). Western blot antibodies for caspase-1 (1:1000, Millipore 06–503 or 06-503-I, 1:1,000, Proteintech 22915-1-AP; 1:2000 Genentech 4175, cell line 4B4.2.1, or 1:1000, AdiPoGen International AG-20B-0042), NLRC4 (1:1000, Millipore, 06–1125), IL-1β (1:1000 R&D Systems and Cell Signaling, AF401-NA and 12426S), IL-18 (1:1000, MBL JM-5180-100), β-actin (1:1000, Sigma A2103), COX2 (1:1000, Cell Signaling 12282), phospho-IκB-α (1:1000, Cell Signaling 9246s), p-ERK (1:400, Cell Signaling 4370), ERK (1:1000, Cell Signaling 9102), IκB-α (1:1000, Cell Signaling 4812), PTGER3 (1:1,000, Abcam ab117998), anti-mouse horseradish peroxidase (HRP), anti-goat HRP, anti-rabbit HRP (1:5000, Abcam ab97046, ab97110 and ab97051, respectively), anti-rat HRP (1:5000 Abcam and Santa Cruz Biotechnology, ab97057 and sc-2006) were used. A rabbit polyclonal antibody raised against *A*. *phagocytophilum* [51] was kindly provided by Erol Fikrig at Yale University School of Medicine (1:2,000). Enhanced chemiluminescence (ECL) western blotting substrate and Super Signal West Pico Chemiluminescent substrate were used (Thermo Scientific). Restore Western Blot Stripping Buffer was used for the stripping of antibodies on the blots (Thermo Scientific).

### ELISA

IL-1β and IL-6 were measured with the BD OptEIA Set (BD Biosciences). IL-18 capture (1:1,000, D047-3) and detection antibodies (1:2,000, D048-6) were purchased from MBL. PGE_2_ was measured with the ELISA kit (Enzo Life Sciences). PGD_2_ was measured with the ELISA kit (Cayman Chemicals). Thromboxane A_2_ was measured with the Mouse Thromboxane A_2_ ELISA Kit (Abbexa).

### Quantitative RT-PCR

Quantitate RT-PCR was performed using the Power SYBR Green PCR Master Mix (Invitrogen) in an ABI 7500 real-time PCR instrument. Primer sequences for *A*. *phagocytophilum* were as follows: 16S-F (5’-CAGCCACACTGGAACTGAGA-3’) and 16S-R (5’-CCCTAAGGCCTTCCTCACTC-3’). Gene expression was normalized by using the primers β-actin-F (5’-ACGCAGAGGGAAATCGTGCGTGAC-3’) and β-actin-R (5’-ACGCGGGAGGAAGAGGATGCGGCAGTG-3’). The absolute quantification method was used. For the PGE_2_-EP3 receptor quantification, PureLink RNA Mini Kit (Invitrogen) and the Verso cDNA synthesis Kit (Thermo Scientific) were used. Gene expression was normalized by using the primers GAPDH-F (5’-TGATGACATCAAGAAGGTGGTGAAG-3’) and GAPDH-R (5’-TCCTTGGAGGCCATGTGGGCCAT-3’). Primer sequences for the EP3 receptor were as follows: EP3-F (5’-GGTTCCTGTGAAGGACTGAAGAC-3’) and EP3-R (5’-AAGGTTCTGAGGCTGGAGATA-3’). The relative quantification method (fold changes) was used.

### Enzymatic assays

15×10^6^ wildtype cells were stimulated with *A*. *phagocytophilum* (MOI 25) overnight. Cells were scraped followed by sonication. COX1/2 enzymatic assays were performed with COX activity assay kit (Cayman Chemicals), whereas cPLA_2_ activity was measured following instructions by the manufacturer (Abnova). Arachidonic acid levels were measured according to the instructions of the ELISA kit (MyBiosource). cAMP was measured by using the cyclic AMP XP Assay Kit (Cell Signaling Technology).

### Illumina sequencing and bioinformatics

BMDMs were grown into 6-well culture plates at 7×10^6^ per well. Cells were stimulated with *A*. *phagocytophilum*. Uninfected BMDMs were used as controls and the experiment was performed in triplicate. Total RNA was isolated with the PureLink RNA Mini Kit (Invitrogen). Illumina Sequencing was performed at the University of Maryland, Baltimore. Briefly, Illumina RNAseq libraries were prepared with the TruSeq RNA Sample Prep kit (Illumina, San Diego, CA). The indexed libraries were pooled and sequenced using the HiSeq platform (Illumina) for the mouse samples in order to generate 101 base pair reads. The reads were further trimmed due to low quality at the trailing 3' end. These trimmed paired end reads were populated into 2 separate FASTQ format files and the quality of the reads was tested using the FastQC toolkit to ensure quality of the sequencing reads.

The RNA sequencing reads were used as input for the TopHat read alignment tool to be aligned to the mouse genomic reference sequence (Ensembl GRCm38 version) for each of the samples. The reference genomic sequences for the GRCm38 genome build were downloaded from the Ensembl resources. The output from TopHat was obtained as BAM format files. In the alignment phase, we allowed up to two mismatches per 30 base pair segment and removed reads that aligned to more than 20 genomic locations. The BAM alignment files obtained from the TopHat alignment tool was analyzed to generate the alignment statistics for each sample, namely, the total number of reads, the number of mapped reads and the percent of mapped reads.

For the differential gene expression analysis, the alignment BAM files from TopHat were further utilized to compute gene expression levels and test each gene for differential expression. The mouse gene set reference annotation (version GRCm38) in GTF format was downloaded from the Ensembl resources. The number of reads that mapped to each gene described in the Ensembl annotation was calculated using the python package HTSeq-an alignment read count tool. The read count represented the expression of the gene. Differential gene expression analysis was conducted using the DESeq R package (available from Bioconductor). The DESeq analysis resulted in the determination of differentially expressed genes. DESeq utilized the read counts provided by the HTSeq read count tool. The read counts for each sample were normalized for sequencing depth and distortion caused by highly differentially expressed genes. The negative binomial model was used to test the significance of differential expression between two genotypes. The differentially expressed genes were deemed significant if the FDR (False Discovery Rate) was less than 0.01, the gene expression was above the 45th percentile and gene showed greater than 2-fold change difference (over expressed or under expressed) between conditions. Principal component analysis and other clustering methods were used to visualize the clustering of the replicates across samples. Heat maps were generated to illustrate the genes showing significant differences between multiple comparisons of the control and other infection and/or treatment conditions.

### *In vivo* infection

C57BL/6 (n = 20) and COX2 (*Ptgs2)* ^*-/-*^ (n = 10) mice were infected by intraperitoneal injection with *A*. *phagocytophilum* strain HZ (1×10^7^ cells). Blood samples were collected at days 0, 5 and 10 for the IL-18 ELISA. Spleens were removed, normalized to the body weight, and compared to those of non-infected mice. Spleens were fixed at day 15 post-infection with 10% neutral buffered formalin and embedded in paraffin wax. Sections (5 μm) were obtained and stained with hematoxylin and eosin. Measurement of *A*. *phagocytophilum* load was done at day 15 post-infection in the peripheral blood of infected animals using quantitative RT-PCR, as described above.

### Statistical analysis

All experiments in this study were performed with at least 2–5 replicates. All data were expressed as means ± standard errors of the means (SEM). The differences between groups were examined by either unpaired Student's *t* test or one-way analysis of variance (ANOVA). All statistical calculations and graphs were made by using GraphPad Prism version 6.0. *P* < 0.05 was considered statistically significant.

## Supporting Information

S1 Fig The *Salmonella*-induced NLRC4 inflammasome is not affected by inhibition of phospholipases. Wildtype (WT) BMDMs (1 x10^6^ cells) were pre-treated for 30 minutes with inhibitors of secreted PLA_2_ (sPLA_2_) (LY315920–10μM), cPLA_2_ (AACOCF3–10μM), phospholipase C (PLC) (U73122–10μM) and phospholipase D (PLD) (FIPI– 0.3μM). Cells were then primed with LPS (50ng/ml) and infected with *Salmonella* (MOI25) for 1 hour. (A) IL-1β, (B) IL-18 and (C) IL-6 were measured in cell culture supernatants by ELISA. (D) SDS-PAGE immunoblot (IB) of caspase-1 p20. (E-G) BMDMs from wildtype (WT) or cPLA_2_-deficient mice (1 x10^6^ cells) were infected with *Salmonella* (MOI25) for 1 hour. Levels of (E) IL-1β and (F) IL-6 were measured in cell culture supernatants by ELISA. (G) SDS-PAGE followed by immunoblot (IB) of caspase-1 p20 in the supernatants. pro-IL-1β and pro-IL-18 detected in lysates. ANOVA-Tukey. *P < 0.05. NS–not significant. (-), non-stimulated.
(TIF)

S2 Fig COX2 does not influence canonical inflammasome activation. BMDMs from wildtype (WT) and COX2 (*Ptgs2*)-deficient mice (1 x10^6^ cells) primed with LPS (50ng/ml) for 1 hour and infected with *Salmonella* (MOI25–1 hour) or stimulated with nigericin (10μM– 18 hours). (A) PGE_2,_ (B) IL-1β**,** (C) IL-18 and (D) IL-6 release in cell culture supernatants was measured by ELISA. (E) Caspase-1 native gel immunoblotting (IB). (F) SDS-PAGE/Western blot indicating caspase-1 autoproteolysis (p20). Student’s t test. *P< 0.05. β-actin and pro-IL-18 used as loading controls.
(TIF)

S3 Fig PGE_2_ does not affect IL-6 secretion during *A*. *phagocytophilum* infection. (A) *Ptgs2* ^-/-^ BMDMs (1 x10^6^ cells) were infected with *A*. *phagocytophilum* for 4 hours followed by addition of PGE_2_ (10 μM), PGD_2_ (10 μM) or TBXA_2_ (10 μM) for 18 hours. IL-6 was measured in the cell culture supernatants by ELISA. (B) Wildtype (WT) BMDMs (1 x10^6^ cells) were pre-treated with the mPGES1 inhibitor CAY10526 at indicated concentrations for 30 minutes followed by *A*. *phagocytophilum* infection (MOI50) for 18 hours. IL-6 was measured in the cell culture supernatants by ELISA. (C) *mPGES1* ^-/-^ BMDMs (1 x10^6^ cells) were infected with *A*. *phagocytophilum* for 4 hours followed by addition of PGE_2_ (10 μM). IL-6 was measured in the cell culture supernatants by ELISA. One-way ANOVA-Tukey. NS, not significant. (-) non-stimulated.
(TIF)

S4 Fig PGE_2_ and IL-18 secretion is dependent on *A*. *phagocytophilum* internalization and RIPK2 function in macrophages. (A) Wildtype (WT) BMDMs (1 x10^6^ cells) were infected with *A*. *phagocytophilum* (MOI50). Cell culture supernatants were collected at indicated time points post-infection. The levels of (A) IL-18 was measured in cell culture supernatants by ELISA. (B) BMDMs from wildtype (WT) and *Ripk2* ^-/-^ mice were infected with *A*. *phagocytophilum* (MOI50) (1 x10^6^ cells) for 18 hours. The levels of IL-18 were measured in cell culture supernatants by ELISA. (C-D) WT BMDMs (1 x10^6^ cells) were pre-treated with indicated concentrations of cytochalasin D for 30 minutes followed by *A*. *phagocytophilum* infection (MOI50) for 18 hours. The levels of (C) PGE_2_ and (D) IL-18 in cell culture supernatants was measured by ELISA. One-way ANOVA-Tukey; Student’s t test. *P < .05. NS, not significant. (-) non-stimulated.
(TIF)

S5 Fig The EP3 receptor modulates NLRC4 inflammasome activity upon *A*. *phagocytophilum* infection. Wildtype (WT) BMDMs (1 x10^6^ cells) were pre-treated for 30 minutes with antagonists of PGE_2_ receptors: (1)–(naïve); (2) (EP1–1μM) (SC51089); (3) (EP2–5μM) (AH6809); (4) (EP3–10μM) (L-798106); (5) (EP4–5μM) (ONO-AE3-208); (6) active (PT– 0.1μg/ml) and (7) catalytically inactive (PT*– 0.1 μg/ml) pertussis toxin and stimulated with (A-D) *A*. *phagocytophilum* (MOI50) for 18 hours. The levels of (A) IL-1β, (B) IL-18 and (C) IL-6 release in cell culture supernatants were measured by ELISA. (D) Caspase-1 autoproteolysis immunoblotting (IB). pro-IL-1β and pro-IL-18 were detected in cell lysates. (E-I) WT BMDMs (1 x10^6^ cells) were pre-treated for 30 minutes with the EP3 antagonist or pertussis toxin at indicated concentrations for 30 minutes followed by *A*. *phagocytophilum* infection (MOI50) for 18 hours. (E, G) IL-18; (F) IL-1β and (H) IL-6 release in cell culture supernatants were measured by ELISA. (I) Caspase-1 autoproteolysis immunoblotting (IB). pro-IL-1β and pro-IL-18 were detected in cell lysates. ANOVA-Tukey. *P < 0.05. NS–not significant. (-), non-stimulated.
(TIF)

S6 Fig The EP3 receptor does not regulate the activity of the canonical NAIP/NLRC4 inflammasome induced by *Salmonella* infection. WT BMDMs (1 x10^6^ cells) primed with LPS (50ng/ml) were pre-treated for 30 minutes with antagonists of PGE_2_ receptors: (1)–(naïve); (2) (EP1–1μM) (SC51089); (3) (EP2–5μM) (AH6809); (4) (EP3–10μM) (L-798106); (5) (EP4–5μM) (ONO-AE3-208); (6) active (PT– 0.1μg/ml) and (7) catalytically inactive (PT*– 0.1 μg/ml) pertussis toxin and stimulated with (A-C) *Salmonella* (MOI25) for 1 hour. The levels of (A) IL-1β and (B) IL-18 release in cell culture supernatants were measured by ELISA. (C) Caspase-1 autoproteolysis immunoblotting (IB). pro-IL-1β and pro-IL-18 were detected in cell lysates. One way ANOVA-Tukey; NS–not significant. (-) non-stimulated.
(TIF)

S7 Fig Membrane-associated adenylyl cyclase modulates the *A*. *phagocytophilum*-induced NLRC4 inflammasome. (A) Wildtype (WT) BMDMs (1 x10^6^ cells) were pre-treated with the EP3 agonist sulprostone (3μM), the EP3 antagonist L-798106 (10μM), or active pertussis toxin (PT– 0.1μg/ml) for 30 minutes followed by *A*. *phagocytophilum* (MOI50) infection for 18 hours. cAMP levels were measured. (B-I) WT BMDMs (1 x10^6^ cells) were pre-treated with the selective (B-E) membrane (Forskolin) or (F-I) soluble (KH7) adenylyl cyclase inhibitors at indicated concentrations for 30 min followed by *A*. *phagocytophilum* colonization (MOI50) for 18 hours. The levels of (B, F) IL-1β, (C, G) IL-18 and (D, H) IL-6 in the cell culture supernatants were measured by ELISA. (E, I) Caspase-1 autoproteolysis was detected with SDS-PAGE immunoblot (IB). Pro-IL-1β and pro-IL-18 were detected in cell lysates. One-way ANOVA-Tukey. *P < 0.05. NS–not significant. (-) non-stimulated.
(TIF)

S8 Fig Schematic representation of the *A*. *phagocytophilum*-induced NLRC4 inflammasome. *A*. *phagocytophilum* infection and formation of the occupied vacuole (ApV) leads to disruption and molecular rearrangements within the cell [28]. (1) Cytosolic phospholipase A2 (cPLA_2_) releases (2) arachidonic acid from phosphatidylinositol 4,5-bisphosphate [PI(_4,5_)P_2_], the major polyphosphoinositide phospholipid present in the inner leaflet of the plasma membrane [52]. (3) Cyclooxygenase 2 (COX2) and microsomal PGE synthase-1 (mPGES1) [29] convert hydrolyzed arachidonic acid to prostanglandin E_2_ (PGE_2_). PGE_2_ exerts its actions by acting on G-protein-coupled receptors (GPCRs). PGE_2_ binds to the EP3 receptor, which inhibits the membrane associated adenylyl cyclase (AC) via Gαi (4). This signaling relay decreases cytosolic cyclic AMP (cAMP) production. Lower levels of cAMP induce the activation of the NLRC4 inflammasome (5). Receptor-interacting serine/threonine-protein kinase 2 (RIPK2) stimulates the production of pro-IL-1β via nuclear factor (NF)-κB signaling (6). RIPK2 also triggers formation of the NLRC4 inflammasome oligomer through COX2 up-regulation (7) via mitogen-activated protein kinase (MAPK) signaling [41]. Caspase-1 cleaves pro-IL-1β and pro-IL-18 leading to the release of mature cytokines (8).
(TIF)
