# Supplementary figures and images for: The Prostaglandin E2-EP3 Receptor Axis Regulates Anaplasma phagocytophilum-Mediated NLRC4 Inflammasome Activation
Source: PLoS Pathog. 2016 Aug 2;12(8):e1005803. doi: 10.1371/journal.ppat.1005803 (PMC4970705; doi:10.1371/journal.ppat.1005803)

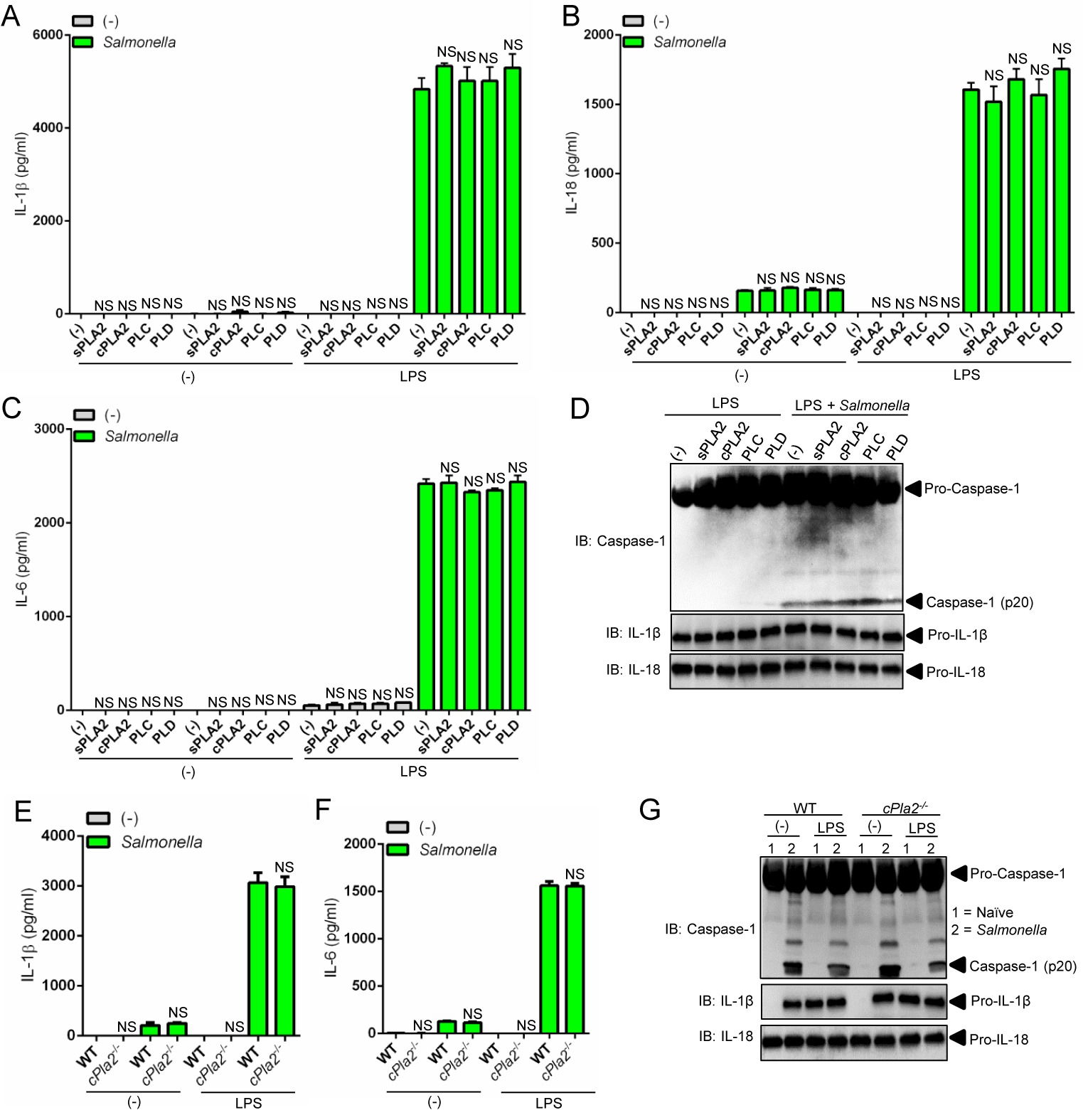

Supplement: S1 Fig — Wildtype (WT) BMDMs (1 x10^6^ cells) were pre-treated for 30 minutes with inhibitors of secreted PLA2 (sPLA2) (LY315920–10μM), cPLA2 (AACOCF3–10μM), phospholipase C (PLC) (U73122–10μM) and phospholipase D (PLD) (FIPI– 0.3μM). Cells were then primed with LPS (50ng/ml) and infected with Salmonella (MOI25) for 1 hour. (A) IL-1β, (B) IL-18 and (C) IL-6 were measured in cell culture supernatants by ELISA. (D) SDS-PAGE immunoblot (IB) of caspase-1 p20. (E-G) BMDMs from wildtype (WT) or cPLA2-deficient mice (1 x10^6^ cells) were infected with Salmonella (MOI25) for 1 hour. Levels of (E) IL-1β and (F) IL-6 were measured in cell culture supernatants by ELISA. (G) SDS-PAGE followed by immunoblot (IB) of caspase-1 p20 in the supernatants. pro-IL-1β and pro-IL-18 detected in lysates. ANOVA-Tukey. *P < 0.05. NS–not significant. (-), non-stimulated. (TIF) [file ppat.1005803.s001.tif]

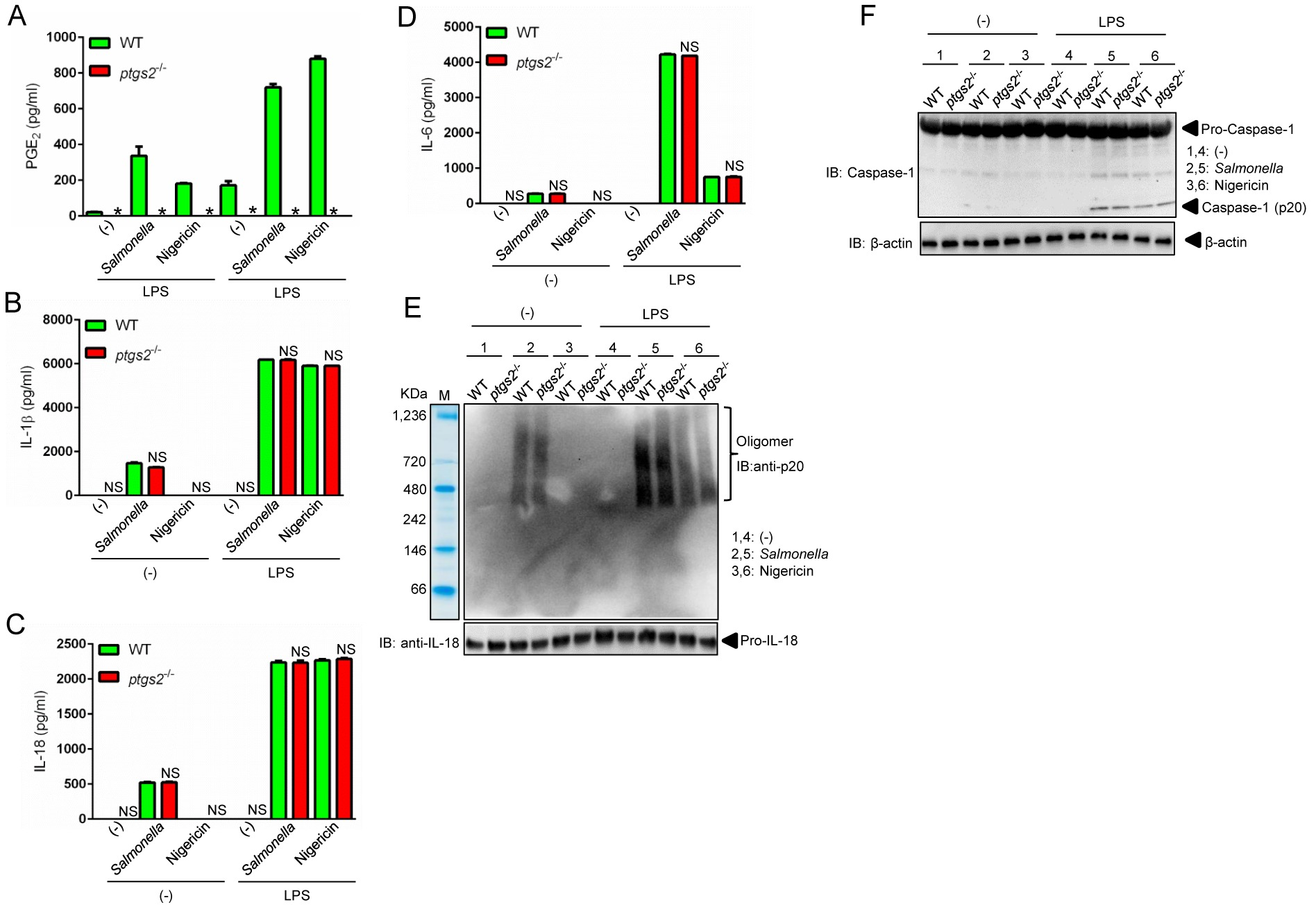

Supplement: S2 Fig — BMDMs from wildtype (WT) and COX2 (Ptgs2)-deficient mice (1 x10^6^ cells) primed with LPS (50ng/ml) for 1 hour and infected with Salmonella (MOI25–1 hour) or stimulated with nigericin (10μM– 18 hours). (A) PGE2, (B) IL-1β, (C) IL-18 and (D) IL-6 release in cell culture supernatants was measured by ELISA. (E) Caspase-1 native gel immunoblotting (IB). (F) SDS-PAGE/Western blot indicating caspase-1 autoproteolysis (p20). Student’s t test. *P< 0.05. β-actin and pro-IL-18 used as loading controls. (TIF) [file ppat.1005803.s002.tif]

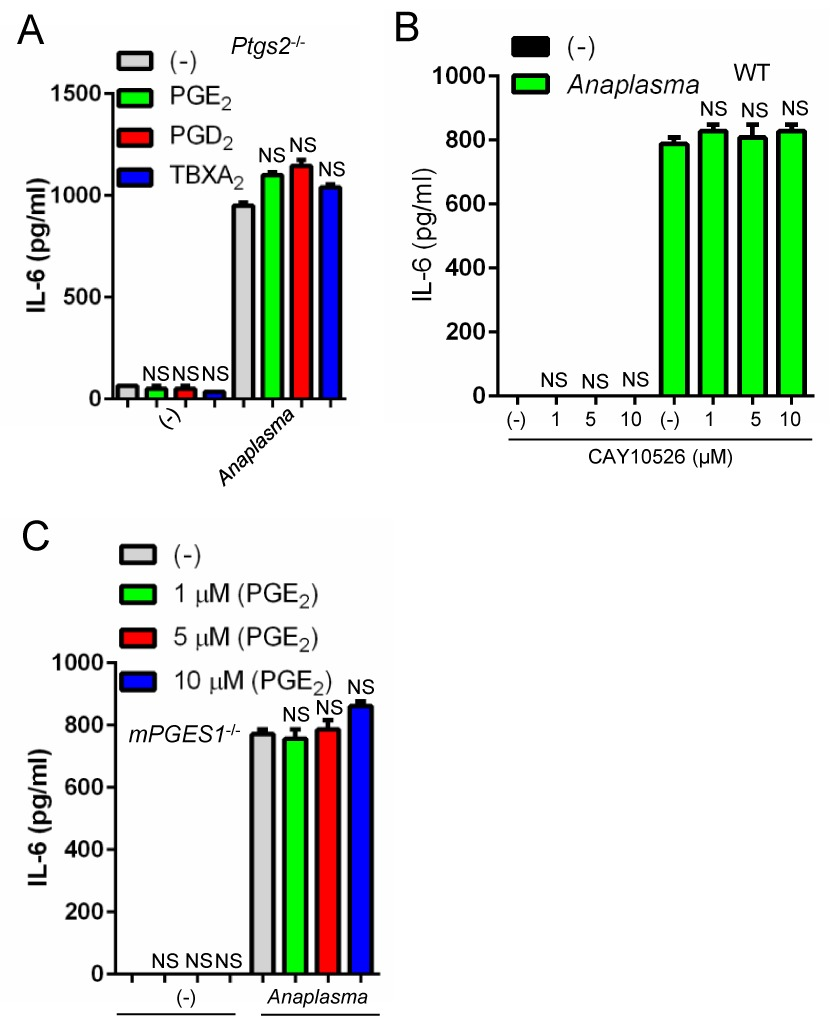

Supplement: S3 Fig — (A) Ptgs2 -/- BMDMs (1 x10^6^ cells) were infected with A. phagocytophilum for 4 hours followed by addition of PGE2 (10 μM), PGD2 (10 μM) or TBXA2 (10 μM) for 18 hours. IL-6 was measured in the cell culture supernatants by ELISA. (B) Wildtype (WT) BMDMs (1 x10^6^ cells) were pre-treated with the mPGES1 inhibitor CAY10526 at indicated concentrations for 30 minutes followed by A. phagocytophilum infection (MOI50) for 18 hours. IL-6 was measured in the cell culture supernatants by ELISA. (C) mPGES1 -/- BMDMs (1 x10^6^ cells) were infected with A. phagocytophilum for 4 hours followed by addition of PGE2 (10 μM). IL-6 was measured in the cell culture supernatants by ELISA. One-way ANOVA-Tukey. NS, not significant. (-) non-stimulated. (TIF) [file ppat.1005803.s003.tif]

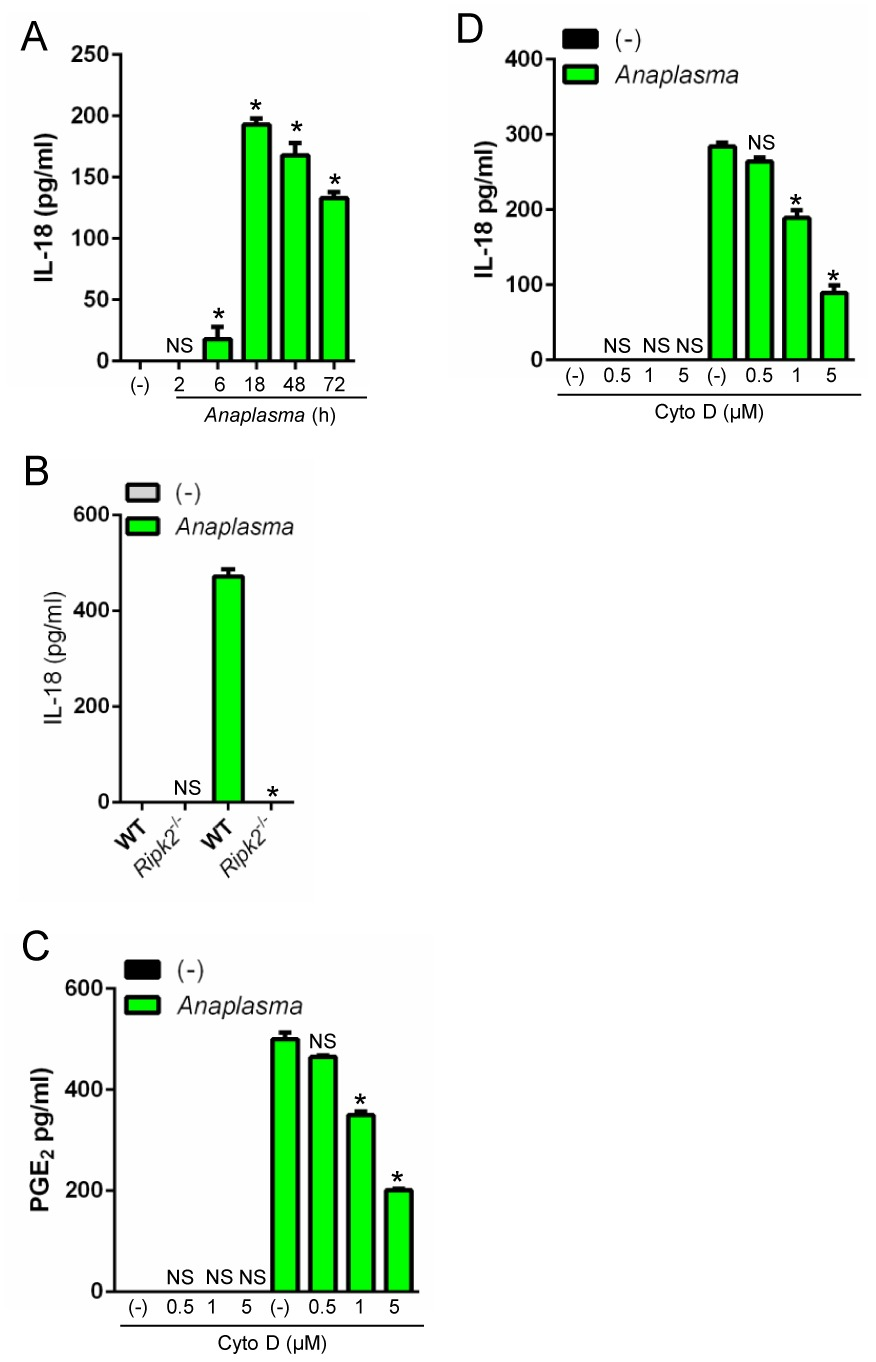

Supplement: S4 Fig — (A) Wildtype (WT) BMDMs (1 x10^6^ cells) were infected with A. phagocytophilum (MOI50). Cell culture supernatants were collected at indicated time points post-infection. The levels of (A) IL-18 was measured in cell culture supernatants by ELISA. (B) BMDMs from wildtype (WT) and Ripk2 -/- mice were infected with A. phagocytophilum (MOI50) (1 x10^6^ cells) for 18 hours. The levels of IL-18 were measured in cell culture supernatants by ELISA. (C-D) WT BMDMs (1 x10^6^ cells) were pre-treated with indicated concentrations of cytochalasin D for 30 minutes followed by A. phagocytophilum infection (MOI50) for 18 hours. The levels of (C) PGE2 and (D) IL-18 in cell culture supernatants was measured by ELISA. One-way ANOVA-Tukey; Student’s t test. *P < .05. NS, not significant. (-) non-stimulated. (TIF) [file ppat.1005803.s004.tif]

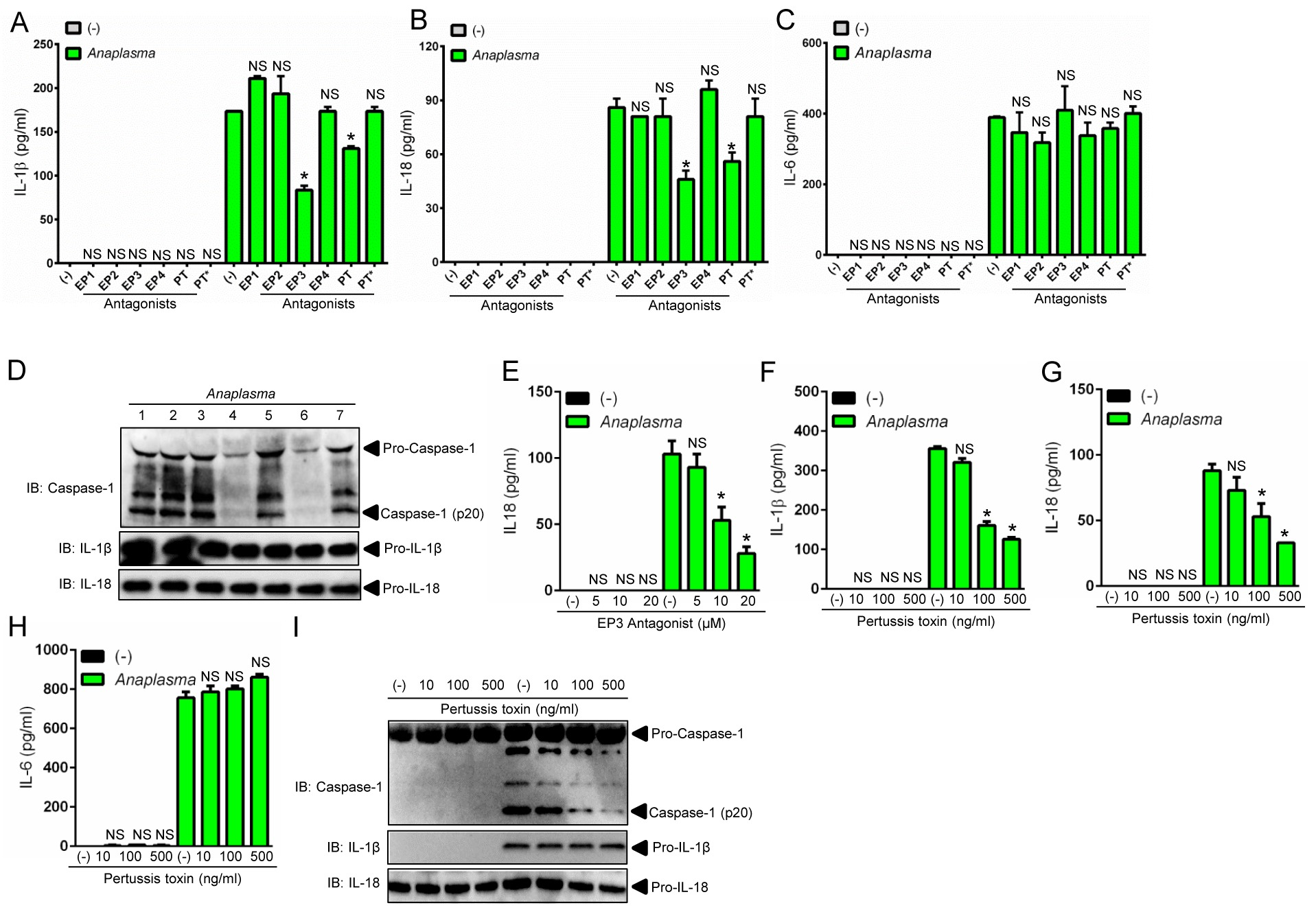

Supplement: S5 Fig — Wildtype (WT) BMDMs (1 x10^6^ cells) were pre-treated for 30 minutes with antagonists of PGE2 receptors: (1)–(naïve); (2) (EP1–1μM) (SC51089); (3) (EP2–5μM) (AH6809); (4) (EP3–10μM) (L-798106); (5) (EP4–5μM) (ONO-AE3-208); (6) active (PT– 0.1μg/ml) and (7) catalytically inactive (PT*– 0.1 μg/ml) pertussis toxin and stimulated with (A-D) A. phagocytophilum (MOI50) for 18 hours. The levels of (A) IL-1β, (B) IL-18 and (C) IL-6 release in cell culture supernatants were measured by ELISA. (D) Caspase-1 autoproteolysis immunoblotting (IB). pro-IL-1β and pro-IL-18 were detected in cell lysates. (E-I) WT BMDMs (1 x10^6^ cells) were pre-treated for 30 minutes with the EP3 antagonist or pertussis toxin at indicated concentrations for 30 minutes followed by A. phagocytophilum infection (MOI50) for 18 hours. (E, G) IL-18; (F) IL-1β and (H) IL-6 release in cell culture supernatants were measured by ELISA. (I) Caspase-1 autoproteolysis immunoblotting (IB). pro-IL-1β and pro-IL-18 were detected in cell lysates. ANOVA-Tukey. *P < 0.05. NS–not significant. (-), non-stimulated. (TIF) [file ppat.1005803.s005.tif]

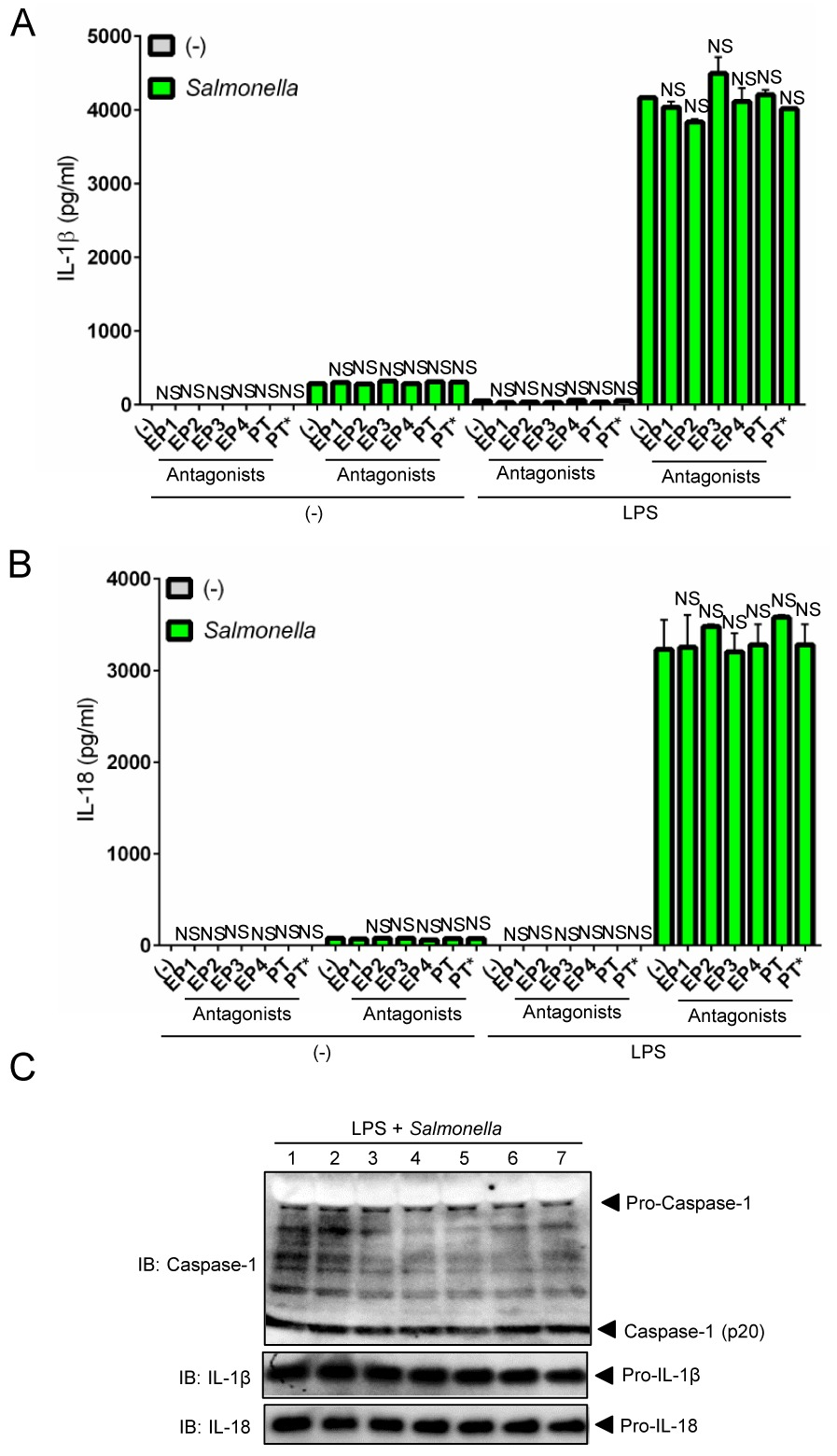

Supplement: S6 Fig — WT BMDMs (1 x10^6^ cells) primed with LPS (50ng/ml) were pre-treated for 30 minutes with antagonists of PGE2 receptors: (1)–(naïve); (2) (EP1–1μM) (SC51089); (3) (EP2–5μM) (AH6809); (4) (EP3–10μM) (L-798106); (5) (EP4–5μM) (ONO-AE3-208); (6) active (PT– 0.1μg/ml) and (7) catalytically inactive (PT*– 0.1 μg/ml) pertussis toxin and stimulated with (A-C) Salmonella (MOI25) for 1 hour. The levels of (A) IL-1β and (B) IL-18 release in cell culture supernatants were measured by ELISA. (C) Caspase-1 autoproteolysis immunoblotting (IB). pro-IL-1β and pro-IL-18 were detected in cell lysates. One way ANOVA-Tukey; NS–not significant. (-) non-stimulated. (TIF) [file ppat.1005803.s006.tif]

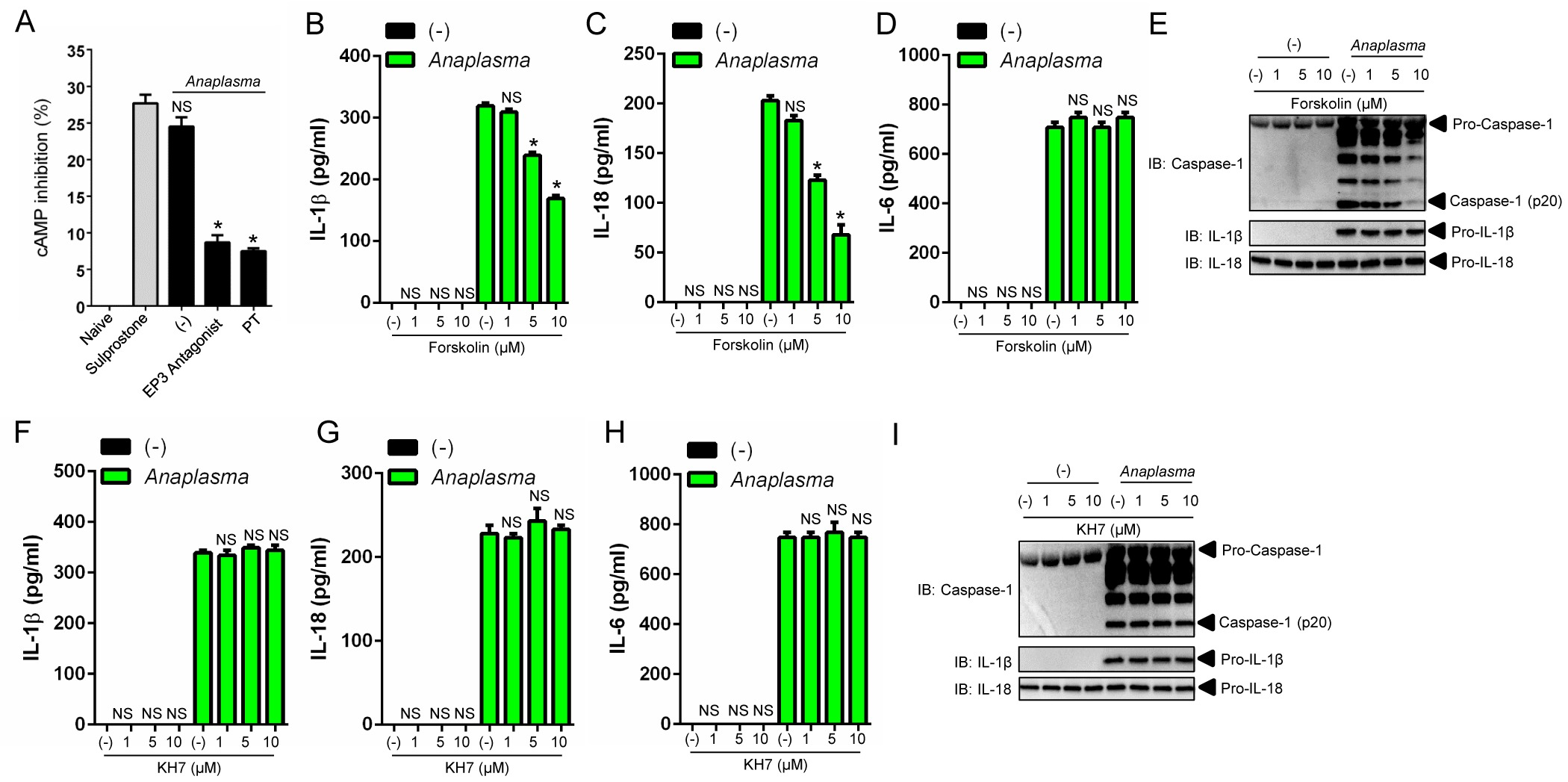

Supplement: S7 Fig — (A) Wildtype (WT) BMDMs (1 x10^6^ cells) were pre-treated with the EP3 agonist sulprostone (3μM), the EP3 antagonist L-798106 (10μM), or active pertussis toxin (PT– 0.1μg/ml) for 30 minutes followed by A. phagocytophilum (MOI50) infection for 18 hours. cAMP levels were measured. (B-I) WT BMDMs (1 x10^6^ cells) were pre-treated with the selective (B-E) membrane (Forskolin) or (F-I) soluble (KH7) adenylyl cyclase inhibitors at indicated concentrations for 30 min followed by A. phagocytophilum colonization (MOI50) for 18 hours. The levels of (B, F) IL-1β, (C, G) IL-18 and (D, H) IL-6 in the cell culture supernatants were measured by ELISA. (E, I) Caspase-1 autoproteolysis was detected with SDS-PAGE immunoblot (IB). Pro-IL-1β and pro-IL-18 were detected in cell lysates. One-way ANOVA-Tukey. *P < 0.05. NS–not significant. (-) non-stimulated. (TIF) [file ppat.1005803.s007.tif]

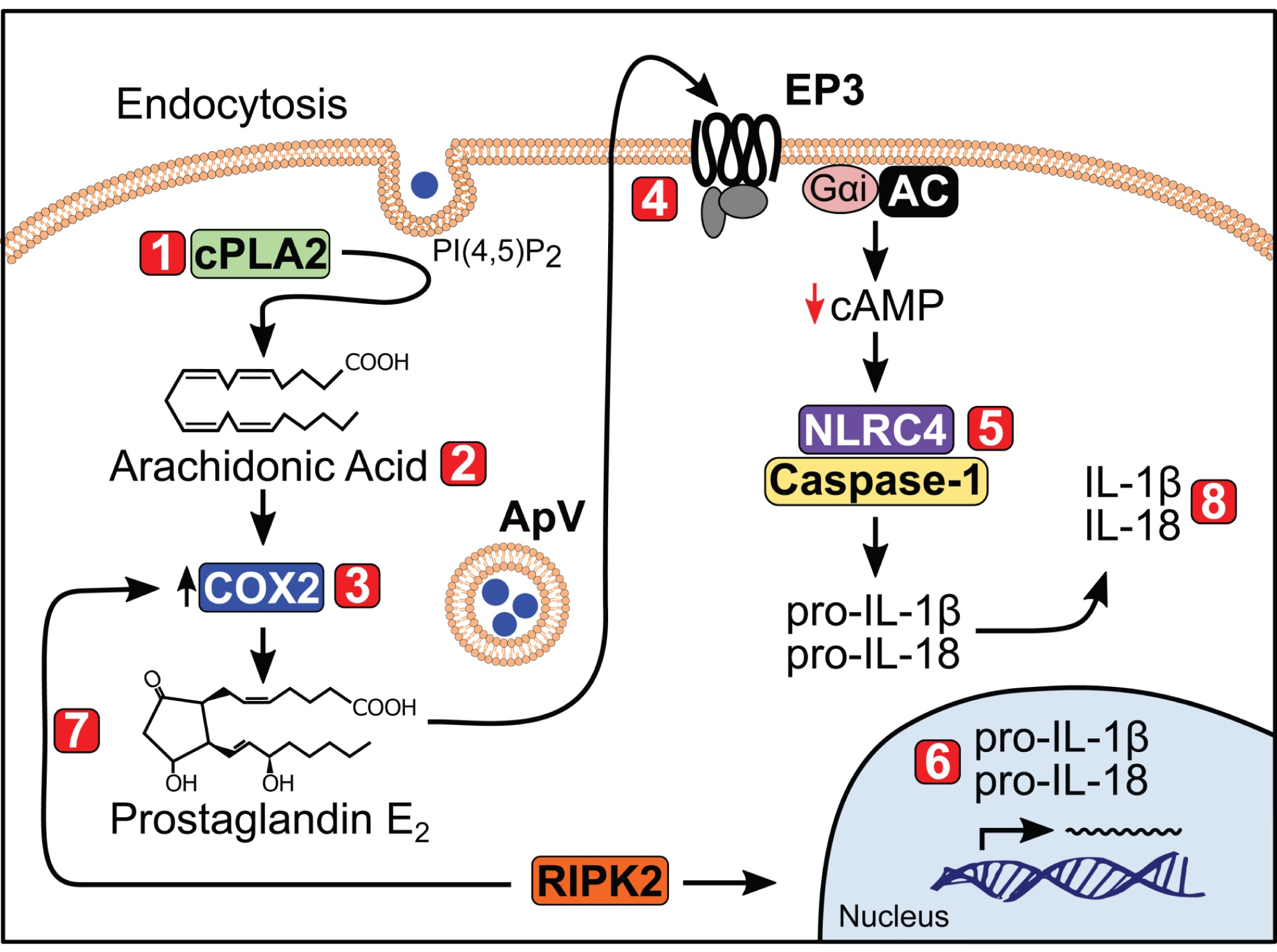

Supplement: S8 Fig — A. phagocytophilum infection and formation of the occupied vacuole (ApV) leads to disruption and molecular rearrangements within the cell [28]. (1) Cytosolic phospholipase A2 (cPLA2) releases (2) arachidonic acid from phosphatidylinositol 4,5-bisphosphate [PI(4,5)P2], the major polyphosphoinositide phospholipid present in the inner leaflet of the plasma membrane [52]. (3) Cyclooxygenase 2 (COX2) and microsomal PGE synthase-1 (mPGES1) [29] convert hydrolyzed arachidonic acid to prostanglandin E2 (PGE2). PGE2 exerts its actions by acting on G-protein-coupled receptors (GPCRs). PGE2 binds to the EP3 receptor, which inhibits the membrane associated adenylyl cyclase (AC) via Gαi (4). This signaling relay decreases cytosolic cyclic AMP (cAMP) production. Lower levels of cAMP induce the activation of the NLRC4 inflammasome (5). Receptor-interacting serine/threonine-protein kinase 2 (RIPK2) stimulates the production of pro-IL-1β via nuclear factor (NF)-κB signaling (6). RIPK2 also triggers formation of the NLRC4 inflammasome oligomer through COX2 up-regulation (7) via mitogen-activated protein kinase (MAPK) signaling [41]. Caspase-1 cleaves pro-IL-1β and pro-IL-18 leading to the release of mature cytokines (8). (TIF) [file ppat.1005803.s008.tif]
